# Dual‐Mode Type I/II Photosensitization of a Stable Mesoporous Hydrogen‐Bonded Organic Framework for Antibacterial Therapy

**DOI:** 10.1002/advs.75173

**Published:** 2026-04-17

**Authors:** Yi‐Lun Cheng, Hui Yuan, Yuhang Li, Zi‐Yu Wang, Rui Wang, Qi Yin, Xue‐Ning Ren, Miao Zheng, Yi Zeng, Tian‐Fu Liu, Zaisheng Ye

**Affiliations:** ^1^ Department of Gastric Surgery, Clinical Oncology School of Fujian Medical University Fujian Cancer Hospital No. 420 Fu‐ma Road Fuzhou Fujian 350014 China; ^2^ University of the Chinese Academy of Sciences Beijing China; ^3^ State Key Laboratory of Structural Chemistry Fujian Institute of Research on the Structure of Matter, Chinese Academy of Sciences Fuzhou Fujian 350002 China; ^4^ Department of Clinical Laboratory Fujian Provincial Maternity and Child Health Hospital Affiliated Hospital of Fujian Medical University Fuzhou Fujian 350001 China; ^5^ College of Chemistry and Materials Science Fujian Normal University Fuzhou 350007 China

**Keywords:** antibacterial, electron donor and acceptor structure, hydrogen‐bonded organic frameworks, Type I and Type II Photosensitizer, wound healing

## Abstract

In contrast to monomers that are randomly distributed in solution or in an amorphous state, constructing a highly crystalline framework with precise molecular arrangement might provide materials with markedly enhanced functionalities. Herein, a large *π*‐conjugated electron donor–acceptor (D–A) type molecule was designed and synthesized, which self‐assembles through hydrogen bonds to form an organic framework named PFC‐513. The ordered arrangement dramatically boosts the quantum yield (ΦΔ) of singlet oxygen generation, classified as type IIreactive oxygen species (ROS), from 0.01 for the monomer to 0.98 for PFC‐513, while enabling the hydroxyl radical generation (type I ROS), which is absent in the monomeric state. Such dual‐mode Type I/II Photosensitization grants PFC‐513 potent antibacterial activity against methicillin‐resistant bacteria and *E. coli*. Beyond direct antibacterial action, PFC‐513 can simultaneously downregulate key pro‐inflammatory cytokines and chemokines under light irradiation for a diabetic wound, therefore synergistically accelerating wound healing. This work establishes a new paradigm for engineering crystalline porous frameworks with superior PDT efficacy, offering a promising strategy for combating infections and promoting wound repair.

## Graphical abstract

A *π*‐conjugated donor–acceptor PDI monomer undergoes hydrogen‐bonded self‐assembly to form a robust mesoporous framework. The ordered molecular arrangement promotes efficient intersystem crossing and charge separation, leading to simultaneous Type I and Type II reactive oxygen species generation with a high singlet oxygen quantum yield. This dual‐mode photodynamic activity confers potent antibacterial effects against MRSA, suppresses pro‐inflammatory cytokine expression, and promotes wound closure in murine models.

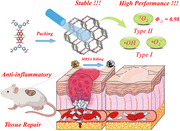

## 1 Introduction

The relentless emergence of antibiotic‐resistant bacteria often leads to life‐threatening complications and poses a formidable threat to global public health, spurring the exploration of alternative antimicrobial strategies. Among them, photodynamic therapy (PDT), employing photosensitizers (PSs) to generate cytotoxic reactive oxygen species (ROS) upon light irradiation, has garnered significant attention as a non‐invasive and minimally resistance‐prone approach [1]. Based on the mechanism of ROS generation, PDT can be classified as Type I or Type II pathway [2]. Type I PDT involves electron‐transfer reactions and produces radical species including superoxide anion •O_2_^−^ and hydroxyl radical •OH, which exhibit potent redox activity and maintain efficacy in hypoxic environments such as those commonly found in infected wounds. However, overproduction of these highly reactive radicals can cause severe collateral damage to host tissues, impairing the healing process [3, 4]. In contrast, Type II PDT operates via energy transfer from photosensitizer to molecular oxygen, generating singlet oxygen (^1^O_2_). This pathway imposes a moderate oxidative stress on surrounding tissues but often suffers from limited efficacy against robust biofilms and deep‐seated infections, as well as the critical dependence on local oxygen concentration [5]. Therefore, the development of photosensitizers capable of simultaneously generating balanced Type I and Type II ROS presents a highly desirable yet challenging strategy to achieve superior antibacterial potency while minimizing adverse side effects.

To address the above issues, self‐assembling electron donor–acceptor (D–A) type molecules with extended *π*‐conjugation into well‐defined porous crystalline frameworks might be a viable strategy [6, 7, 8, 9, 10, 11, 12]. The ordered arrangement of donor‐acceptor motifs facilitates electron transfer, which promotes the Type I ROS generation [13]. Meanwhile, the extended *π*‐conjugation resulted in prolonged triplet‐state lifetime, facilitating energy transfer for Type II ROS production [13]. Furthermore, aggregating monomers into infinite ordered supramolecular arrays results in broadened energy bands, which reduce the singlet‐triplet energy gap (ΔE_ST_) [14]. The narrowed ΔE_ST_ means the close proximity of excited‐state energy levels, which not only promotes the original S_1_→T_1_ intersystem crossing (ISC) but also opens additional ISC channels from singlet to nearby triplet states [15]. Consequently, both Type I and Type II photoreactions are enhanced and lead to superior ROS generation. More strikingly, the generated porous matrix promotes efficient oxygen diffusion and offers confined space for therapeutic agent loading, significantly broadening its potential for combined antimicrobial therapies [16, 17].

Herein, we design a D–A type monomer with large *π*‐conjugation and assemble molecules into a stable mesoporous hydrogen‐bonded organic framework, termed PFC‐513 (PFC stands for Porous materials from FJIRSM, CAS). The ordered molecular aggregation brings material exceptional high singlet oxygen quantum yield (ΦΔ= 0.98) compared with its monomer (barely observed), ranking among the highest reports for crystalline organic materials [18]. More importantly, type I ROS (•OH and •O_2_^−^) were efficiently generated which cannot achieve for its monomeric state. The simultaneously generating both type I and type II ROS can be attributed to the well‐ordered and extended *π*‐conjugated donor‐acceptor (D‐A) structure, which promotes efficient charge separation, reduces the singlet‐triplet energy gap, and enables simultaneous electron and energy transfer pathways under photoexcitation. The followed in vitro experiments demonstrate that PFC‐513 exerts potent antibacterial PDT activity against methicillin‐resistant MRSA and *E. coli*. For refractory MRSA‐infected diabetic wounds, it's striking to find that the antibacterial PDT also significantly downregulate key pro‐inflammatory cytokines and chemokines, and modulate critical signaling pathways including NF‐κB and TNF‐α according to the transcriptomic analysis. The suppression of excessive inflammation reduces immune‐mediated tissue damage and provide regenerative microenvironment, which synergistically accelerate diabetic wound closure and tissue repair. This work not only introduces a superior HOF‐based photosensitizer but also establishes a new design paradigm for developing multimodal photocatalytic platforms for advanced antimicrobial therapy.

## 2 Results and Discussion

### 2.1 Synthesis and Characterizations of PFC‐513

**FIGURE 1:**
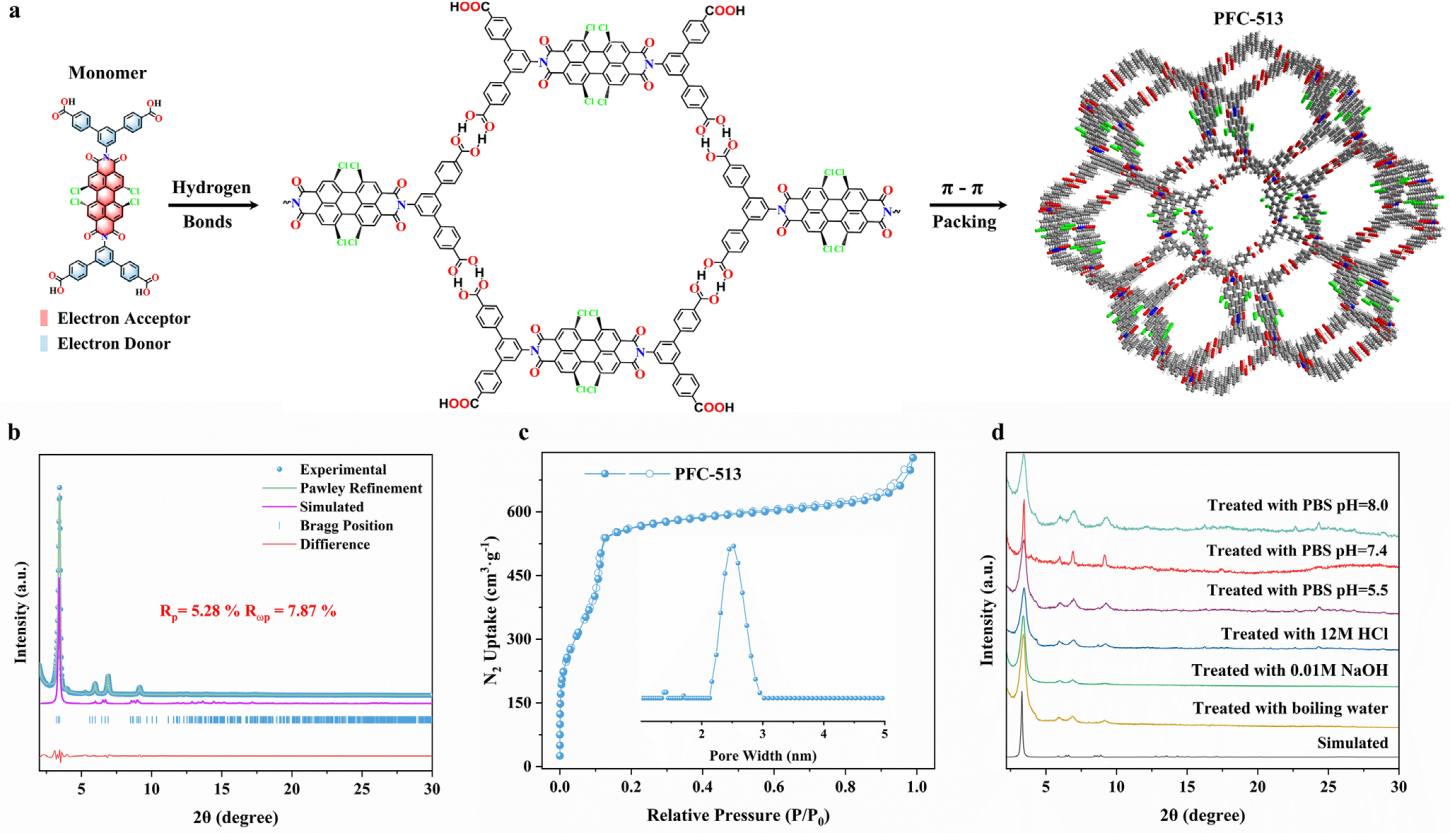
(a) The chemical structure of N,N′‐di(3′,3″,5′,5″‐tetrakis(4‐carboxyphenyl))‐1,2,6,7‐tetrachloroperylene‐3,4,9,10‐tetracarboxylic acid diimide and the schematic representation of PFC‐513 crystal structure. (b) The experimental and simulated PXRD patterns of PFC‐513. (c) N_2_ sorption isotherm of PFC‐513 at 77 K and pore size analysis of PFC‐513. (d) The PXRD pattern of PFC‐513 treated various solutions.

**FIGURE 2:**
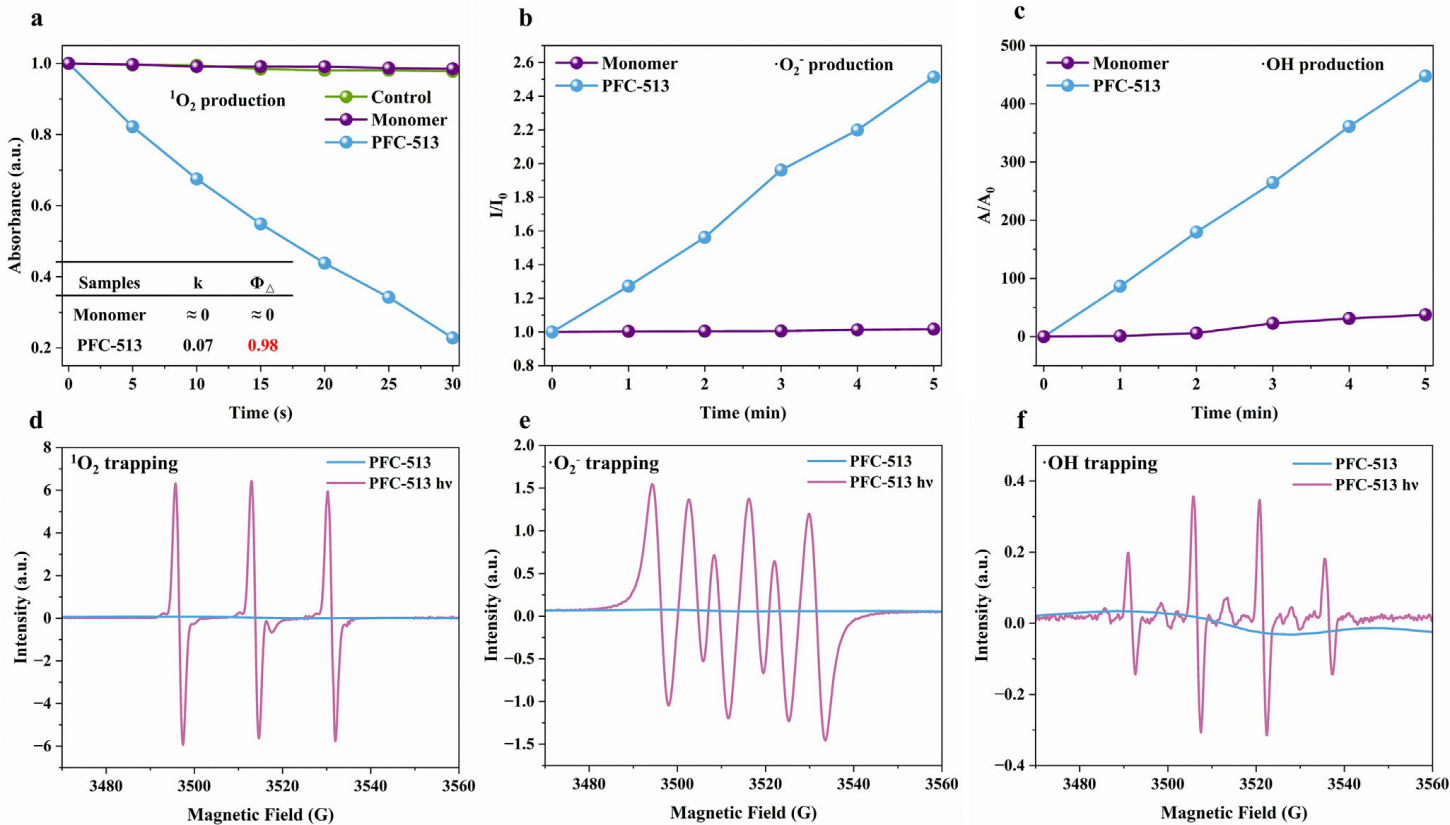
(a) Singlet oxygen yields of PFC‐513 and monomer with respect to RB. (b) Fluorescence intensity of Dihydrorhodamine 123 (DHR123) over irradiation time in the presence of PFC‐513 and monomer. (c) Absorbance of Salicylic Acid (SA) over irradiation time in the presence of PFC‐513 and monomer (d) ESR spectral studies showing O_2_^•−^ generation by PFC‐513 (1 mg/mL) under light irradiation; 2,2,6,6‐Tetramethyl‐4‐piperidine (TEMP) was used as trapping agent for ^1^O_2_, 5,5‐dimethyl‐1‐pyrroline N‐oxide (DMPO) was used as trapping agent for (e) O_2_^•−^ and (f) OH.

The structure of PFC‐513 was simulated using the Materials Studio software package based on its isostructural model of PFC‐55 [19], in which each molecule was connected through hydrogen bonds and form one‐dimension honey‐comb channels in the (100) direction (Figure 1a; Figure S9). Pawley refinement of the experimental powder X‐ray diffraction (PXRD) patterns based on the simulated unit cell parameter gave rise to an excellent agreement factor and a low residual value (R_p_ = 5.28% and R_wp_ = 7.87%) (Figure 2b). High‐resolution transmission electron microscopy (HRTEM) images clearly showed a lattice fringe spacing of 2.5 nm, which can be assigned to the (011) plane (Figure S12). Fourier‐transform infrared spectroscopy (FT‐IR) confirmed the presence of well‐matched chemical bonds (Figure S26). These results collectively validate the structure model of PFC‐513. Nitrogen isotherm showed PFC‐513 featured a typically type IV adsorption behavior with a steep increase before P/P_0_ = 0.2, indicating its mesoporous characteristic, which is relatively rare in previously reported hydrogen‐bonded organic frameworks [17]. Brunauer–Emmett–Teller (BET) analysis revealed the specific surface area of 1786.9 m^2^/g and the pore size was calculated to be 2.51 nm (Figure 1c; Figure S9), which is in good agreement with the crystallographic result. PFC‐513 maintains its crystallinity both upon immersion in various solvents for one week and upon heating to 573.15 K as confirmed by PXRD and N_2_ sorption. (Figure 1d; Figures S3–S8). Thus, these results demonstrate the remarkable chemical and thermal stability of PFC‐513. Moreover, PFC‐513 can form a colloid and exhibit unique solution processibility, which can be confirmed by the distinct Tyndall effect (Figure S10), thereby enabling the fabrication of PFC‐513‐based hydrogel for the potential use as a wound dressing. (Figure S11).

### 2.2 ROS Generation and Characterization

**FIGURE 3:**
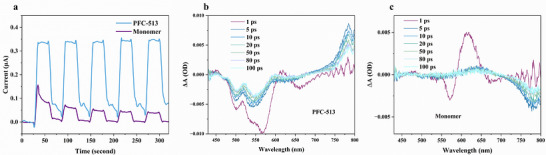
(a) Transient photocurrent responses of PFC‐513 and its monomer in 0.2 M Na_2_SO_4_ aqueous solution under visible light irradiation. Femtosecond transient absorption spectra of (b) PFC‐513 and (c) monomer suspended in ethanol excited upon a 365 nm laser pulse.

In type I PDT, the photosensitizer generates •OH and •O_2_^−^ (as intermediates) via oxygen‐independent electron transfer cascade redox reactions [20]. Therefore, the Type I pathway is a viable option for treating hypoxic deep wounds [2]. Electron paramagnetic resonance (EPR) spectroscopy using DMPO as a spin trap revealed dramatically enhanced signals for both •OH and •O_2_^−^ under Xe lamp irradiation, confirming efficient Type I ROS generation by PFC‐513 (Figure 2e,f). Electrostatic potential (ESP) calculations confirm the electron D‐A configuration in the monomer (Figure S29). To further reveal how the molecular aggregation promotes ROS generation, •OH and •O_2_^−^ production of monomer and PFC‐513 were quantitatively determined using salicylic acid and dihydrorhodamine 123 as probes, respectively (Figure 2b,c). Notably, PFC‐513 exhibited a 450‐fold enhancement in •OH generation and a 2.4‐fold increase in •O_2_^−^ production compared to its monomer, unequivocally demonstrating its superior radical generation efficiency (Figure S23). To further elucidate the behind mechanism, the redox potential of PFC‐513 was investigated by cyclic voltammetry. As shown in Figure S25a, the ground‐state reduction potential E_red_ of PFC‐513 is ‐0.78 V (vs. NHE), which is lower than the reduction potential of O_2_ (E_red_ (O_2_/ •O_2_^−^) = −0.33 V vs. NHE) and therefore is capable to reduce oxygen to superoxide anions [21]. Photocurrent tests and Electrochemical impedance spectroscopy (EIS) verified that the molecular *π*–*π* packing in PFC‐513 enhances the charge‐separation ability and lowers charge transfer resistance compared to its monomer, thus facilitating the generation of type I ROS through electron transport (Figure 3a; Figure S18). To evaluate singlet oxygen production through the type II pathway, we used 1,3‐diphenylisobenzofuran (DPBF) as an indicator irradiated by a 525 nm (1.5 W/cm^2^) laser (Figure 2a) [22]. It shows that 0.5 mg/mL PFC‐513 achieved about 100% degradation of the DPBF solution in 15 s under the 525 nm light irradiation (Figure S22), and the generation of ^1^O_2_ was confirmed by the characteristic 1:1:1 peak in EPR spectroscopy with TEMP as a trapping agent (Figure 2d). Conversely, no detectable ^1^O_2_ signal was observed under dark conditions. In addition, the absorbance of DPBF almost shows no degradation in the presence of PFC‐513 or without photoirradiation, suggesting the photodynamic crucial role in the photocatalytic reaction. The singlet oxygen (^1^O_2_) quantum yields of PFC‐513 and monomer were determined to be 0.98 and trace amount, respectively, with Rhodamine B (RB) as a standard (Figure 2a; Table S3) [23].

### 2.3 Photophysical Properties and Mechanism

To gain insight into the mechanism of the simultaneous Type I and Type II ROS production of PFC‐513, steady‐state photoluminescence studies were conducted. Both PFC‐513 and its monomer exhibit moderate fluorescence intensity and relatively short lifetime (0.39 and 0.32 ns, respectively) (Figure S19). Photothermal conversion of PFC‐513 and monomer were determined to be 3.8% and 8.8%, respectively (Figure S30 and Table S4). The low fluorescence emission intensity and photothermal conversion efficiency of PFC‐513 implied that many excitons might release their energy through the ISC pathway. Temperature‐dependent PL spectroscopies were also measured to further explore the exciton behavior of PFC‐513. The exciton binding energy (E_b_) was obtained by fitting the integrated PL peak intensity using the Arrhenius equation, I(T)  =  I0 /(1  +  Aexp(‐Eb/kBT)). It is shown that the E_b_ of PFC‐513 (43.0 eV) was smaller than that of the monomer (50.0 eV) (Figure S20), which is attributed to the extended *π*‐conjugated structure and enhanced electron delocalization in PFC‐513, effectively reducing the coulombic interaction between the electron and hole [24]. Furthermore, EPR spectroscopy revealed a remarkably enhanced single‐electron signal for PFC‐513 upon illumination compared to its monomer, confirming the highly delocalized nature of its photoexcited state (Figure S21) [25]. To further disclose the exciton behaviors in PFC‐513, femtosecond transient absorption (TA) spectra were also employed (Figure 3b,c; Table S1). Upon excited by a 365 nm laser, PFC‐513 exhibited two negative and strong signals at 500 and 550 nm. Based on UV–vis and fluorescence spectra, these two signals can be attributed to the ground‐state bleach (GSB) and stimulated emission (SE), respectively. Additionally, the positive signals at 725–800 nm appeared, which can be assigned to the excited state absorption (ESA). As the signal at the region of 725–800 nm gradually decreased to zero after 50 ps, the characteristic absorption peaks at 500 and 550 nm are retained. In addition, these negative absorption bands appeared, and the intensity kept almost the same after 50 ps, indicating the formation of a long‐lived excitation state in PFC‐513 [26]. Combining the above results, we speculate that the transition process of singlet exciton to triplet exciton efficiently occurred under photoirradiation in PFC‐513. In sharp contrast to PFC‐513, the TA spectra of monomer showed a week GSB and SE centered at 500 nm and a weak ESA between 730 and 780 nm [18]. The above results show that the ordered molecular aggregation through *π*–*π* stacking is conducive to the population of the triplet state, thereby promoting the ^1^O_2_ generation through a triplet‐triplet energy transfer process. Therefore, it is reasonable to conclude that PFC‐513 efficiently dissipate the harvested photon energy via nonradiative relaxation pathways, thereby generating ROS for highly efficient photodynamic therapy.

### 2.4 Antibacterial Properties

**FIGURE 4:**
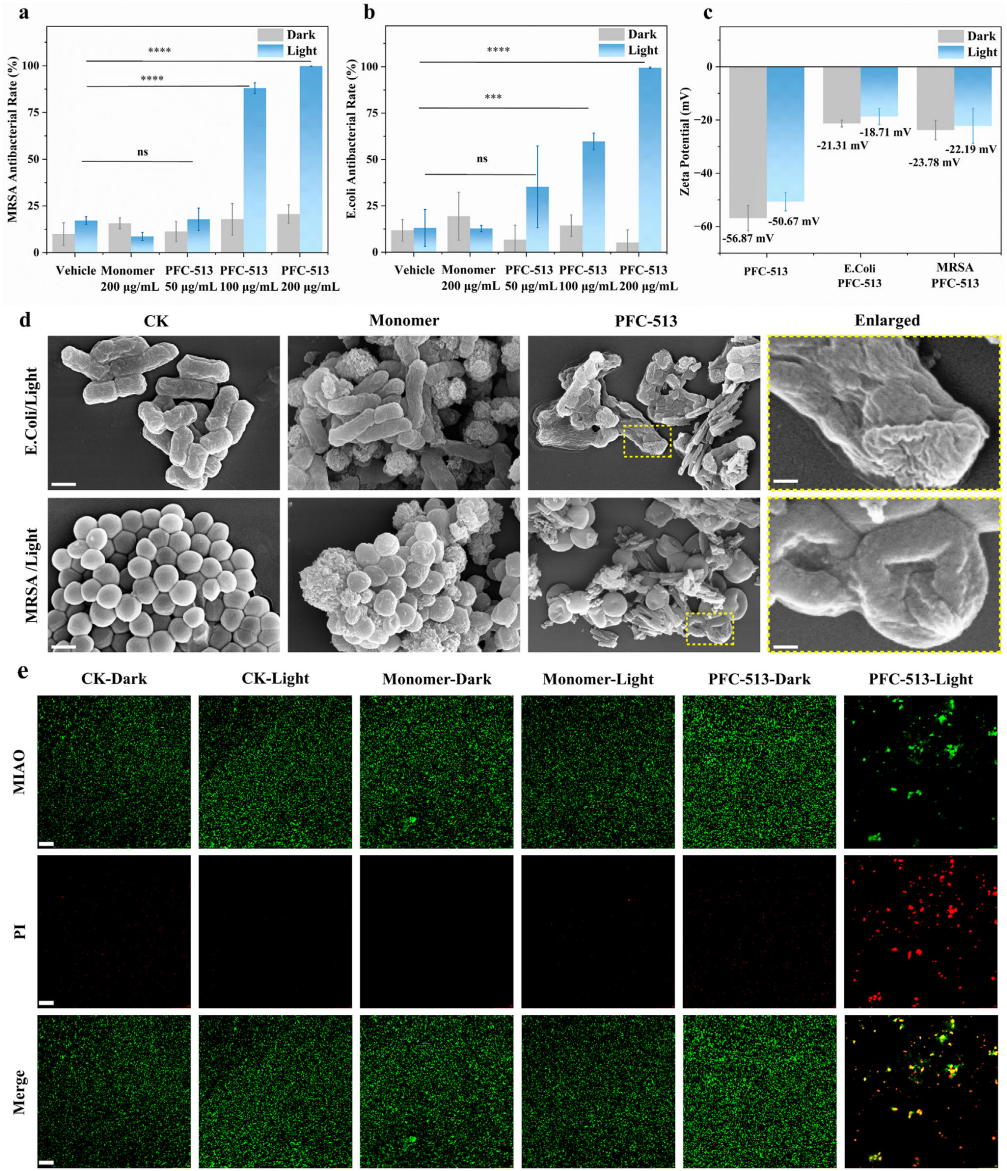
(a) MRSA antibacterial rates in the presence of PFC‐513 and monomer. (b) *E. coli* antibacterial rates in the presence of PFC‐513 and monomer. (c) The Zeta Potential of PFC‐513 co‐treated with *E. coli* and MRSA. (d) SEM images of *E. coli* and MRSA (scale bar: 1 µm, 200 nm). (e) Live/Dead staining images of MRSA (scale bar: 40 µm). Statistical data are presented as mean ± SD (^*^ *p <* 0.05, ^**^ *p <* 0.01, ^***^ *p <* 0.001, ^****^ *p <* 0.0001).

Inspired by the efficient dual‐mode ROS production, we evaluated its antibacterial efficacy against methicillin‐resistant *Staphylococcus aureus* (MRSA) and *Escherichia coli* (E. coli). Quantitative analysis of colony‐forming units (CFUs) revealed potent light‐dependent bactericidal activity for PFC‐513 (Figure 4a,b), showing greater than 90% eradication for both MRSA and E. coli compared to the saline control group. In contrast, negligible antibacterial activity (colony reduction <15%) was observed for PFC‐513 in the dark, confirming its PDT activity. Furthermore, monomer groups exhibited no antibacterial effect under either light or dark conditions (Figure S31). Notably, upon incubation with *E. coli* and *S. aureus*, the zeta potential of PFC‐513 exhibited a significant shift, indicating a strong interaction between the material and the bacterial surface (Figure 4c). Further morphological evaluation of the light‐treated bacteria was conducted using SEM and TEM. As shown in Figure 4d and Figure S32, the light‐treated bacteria exhibited severe morphological damage with membrane rupture, surface collapse, and leakage of cytoplasmic contents, indicating a complete loss of structural integrity [27]. In contrast, neither PFC‐513 in dark nor monomer‐treated (with or without light) groups showed structural damage. Confocal microscopy studies of Live/Dead staining revealed distinct red fluorescence in both MRSA and E. coli after being treated with PFC‐513 under light irradiation, indicating extensive bacterial death, while the control groups exhibited green fluorescence characteristic of viable bacterial (Figure 4e; Figure S34), indicating the significant PDT activity of PFC‐513.

### 2.5 Diabetic Wound Healing Performance

**FIGURE 5:**
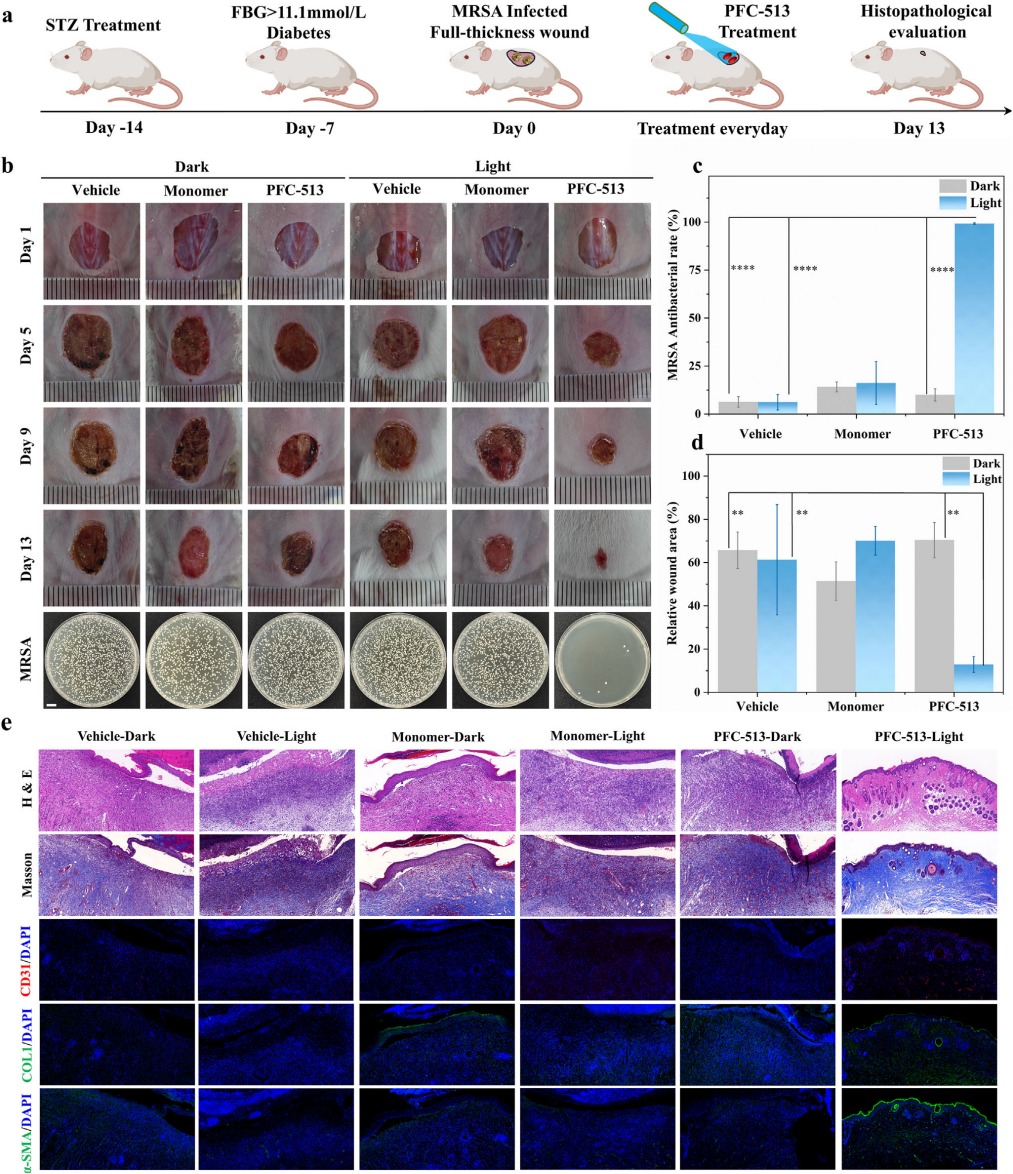
(a) Schematic diagram of the PFC‐513‐based treatment strategy for infected diabetic full‐thickness skin wounds, FBG: fasting blood glucose. (b) Representative images of wounds treated with various materials at 1, 5, 9, and 13 days and photographs of MRSA spread plates from skin samples (Scale bar: 1 mm). (c) MRSA antibacterial rates in the wound. (d) Statistics of remaining wound area (*n* = 5). (e) Hematoxylin & eosin (H&E), Masson trichrome, COL1, CD31, and 𝛼‐SMA staining images of wound healing. (Scale bar: 1 mm). All data are presented as mean ± SD (^*^ *p <* 0.05, ^**^ *p <* 0.01, ^***^ *p <* 0.001, ^****^ *p <* 0.0001), and statistical significance was analyzed using one‐way ANOVA.

To further demonstrate its therapeutic potential of this material against bacterial infection‐induced inflammatory, we used a MRSA‐infected diabetic wound mouse model to evaluate its therapeutic effect. Results showed that a significantly reduced wound area was observed for the light‐activated PFC‐513 group compared to all the other control groups after 13 days’ treatment (Figure 5b,d). To evaluate the in vivo antibacterial efficacy of PDT treatment, wound exudates were collected on day 5 for bacterial culture analysis. The results revealed that the PFC‐513 plus light irradiation group markedly suppressed bacterial growth, achieving an antibacterial efficiency of approximately 99.24% (Figure 5c). In stark contrast, other groups exhibited negligible antibacterial activity and consequently delayed wound healing. Figure 5e illustrates markedly enhanced wound healing following PFC‐513‐mediated PDT, as evidenced by H&E staining on day 13. The PFC‐513+light group exhibited substantial granulation tissue formation, accelerated neovascularization, thickened neo‐epidermis, and reduced inflammatory cell infiltration [28]. Masson's staining further demonstrated improved collagen deposition and organization, which are essential for restoring mechanical strength and integrity in the regenerated tissue (Figure 5e; Figure S40) [29]. Immunofluorescence revealed significantly enhanced CD31^+^ intensity (Figure S41) and a 1.5‐fold increase in COL1A1 (Figure 5e; Figure S42) deposition in wounds after PDT treatment of PFC‐513, indicating promoted angiogenesis and collagen production. Furthermore, significant upregulation of α‐SMA^+^ myofibroblasts was also uniquely observed in this group, confirming improved matrix remodeling and contraction (Figure S43). These results demonstrate that PDT delivered by PFC‐513 accelerates wound healing through synergistic angiogenesis, collagen deposition, and myofibroblast‐driven contraction.

### 2.6 Transcriptomic Insights: From Bactericidal Effects to Anti‐Inflammatory Regulation

**FIGURE 6:**
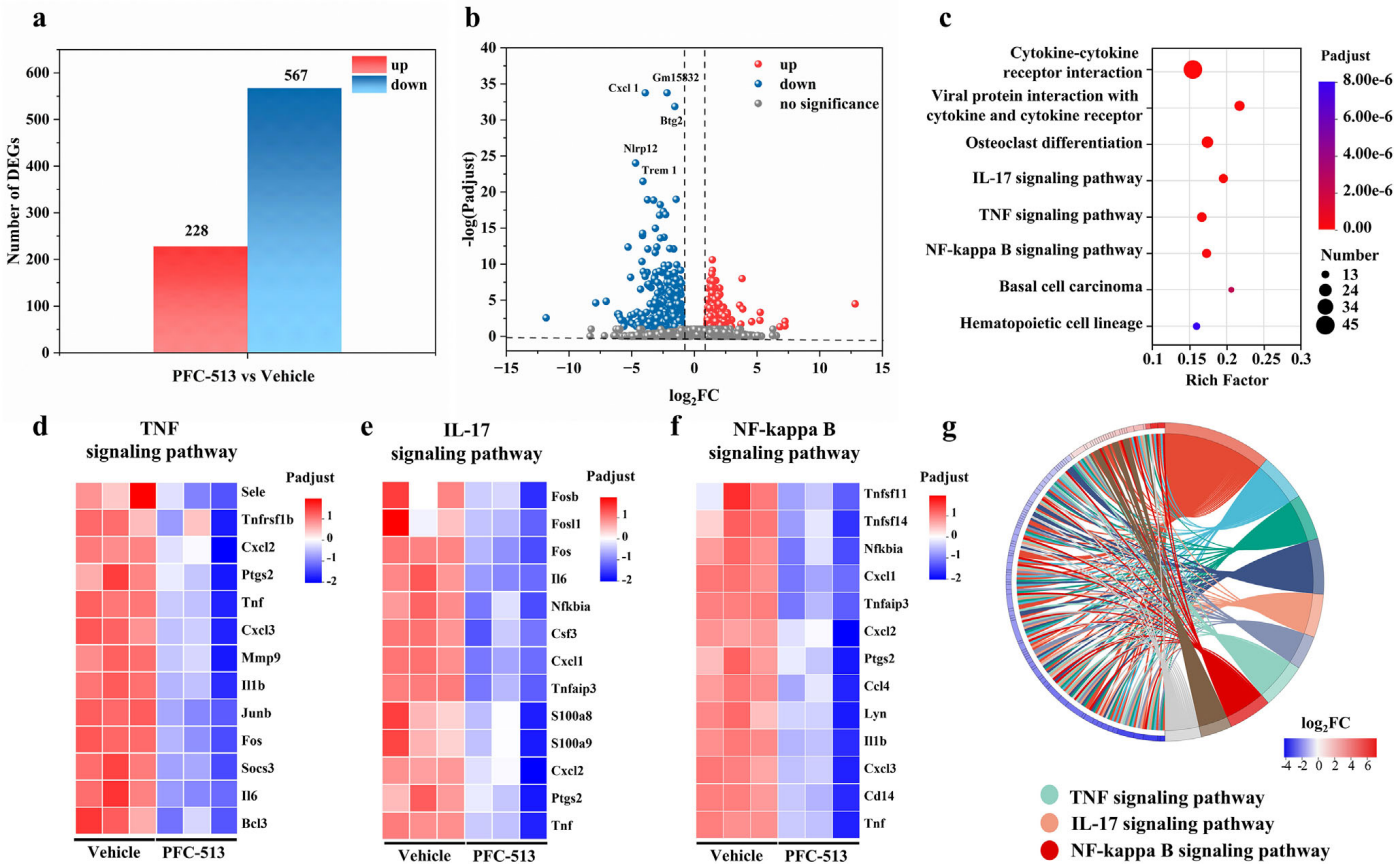
RNA‐sequence analysis of wound tissue after being treated with PFC‐513 under light irradiation. (a) Numbers and (b) volcano plots of up‐regulated and down‐regulated genes of PFC‐513 under light irradiation groups compared to vehicle groups (*n* = 3). (c) KEGG pathway enrichment analysis. (d) Heat maps of TNF signaling pathway genes. (e) Heat maps of the IL‐17 signaling pathway. (f) Heat maps of NF‐kappa B signaling pathway genes. (g) Circular visualization of KEGG enrichment analysis of inflammation related DEGs.

Transcriptome analysis was performed on the collected wound tissue samples to elucidate how the PDT treatment enhances the wound healing of mice [30]. As shown in Figure 6a, RNA‐sequence analysis identified 795 differentially expressed genes (DEGs) in the PFC‐513 PDT‐treated group compared to the vehicle control, consisting of 228 (28.68%) downregulated and 567 (71.32%) upregulated genes. To further elucidate the mechanism, we examined specific genes involved in inflammatory responses and bactericidal effects. Treatment with PDT PFC‐513 significantly downregulated the transcription of key players in wound healing, including pro‐inflammatory cytokines (e.g., IL‐17) and chemokines (Cxcl1) (Figure 6b). In Figure 6c, Kyoto Encyclopedia of Genes and Genomes (KEGG) pathway analysis revealed significant downregulation of pro‐inflammatory cytokine and chemokines genes within key pathways, including cytokine‐cytokine receptor interaction pathway, NF‐κB signaling, TNF signaling, and IL‐17 signaling pathways (Figure 6d–g). Gene Ontology (GO) analysis reveals that PFC‐513 PDT significantly modulates genes enriched in inflammatory response, cell migration, and bacterial response. (Figure S44). Bacterial lipopolysaccharide (LPS) is usually considered as the major factor impeding wound repair through activating NF‐κB signaling and instigating a detrimental inflammatory cascade [30]. We proposed that PFC‐513 PDT attenuates this pathological response through reducing pro‐inflammatory factor release and inhibiting the NF‐κB, TNF, and IL‐17 signaling pathways [31, 32], therefore promoting the healing process.

## 3 Conclusions

In conclusion, we developed a stable mesoporous HOF PFC‐513 through self‐assembling large *π*‐conjugated electron D‐A monomer, which shows excellent photodynamic activity capable of simultaneously generating both type I and type II reactive oxygen species (ROS). As a result, this material exhibits potent, synergistic antibacterial activity against challenging pathogens like MRSA and E. coli in a diabetic wound. It's striking to find that PFC‐513 can simultaneously confer a notable anti‐inflammatory effect by modulating the associated inflammatory pathways, therefore, an accelerated wound healing effect was observed. This study presents a new paradigm for designing porous organic materials as multifunctional and highly efficient photosensitizers for antimicrobial therapy and wound management.

## Author Contributions

Y.L.C. performed on the design and results analysis of all experiments, and wrote the manuscript. H.Y. assisted in the animal experiment. Y.H.L. revised the manuscript and advised on the design of animal experiments. Z.Y.W. and R.W. performed materials characterization. Z.S.Y, Y.Z., and M.Z. designed and analyzed all the animal experiments. Q.Y. helped the structure simulated. X.N.R performed SEM characterization. T.F.L. guided the material design and synthesis part of this work.

## Conflicts of Interest

The authors declare no conflicts of interest.

## Supporting information




**Supporting File**: advs75173‐sup‐0001‐SuppMat.pdf.

## Data Availability

The experimental data generated in this study are provided within the Article, Supplementary Information, and Source Data file. All data are available from the corresponding authors upon request.
