# Supplementary material for: Dual‐Mode Type I/II Photosensitization of a Stable Mesoporous Hydrogen‐Bonded Organic Framework for Antibacterial Therapy
Source: Adv Sci (Weinh). 2026 Apr 17;13(36):e75173. doi: 10.1002/advs.75173 (PMC13317695; doi:10.1002/advs.75173)
Supplement: Supplementary file 1 — Supporting File: advs75173‐sup‐0001‐SuppMat.pdf. [file ADVS-13-e75173-s001.pdf]

Supporting Information

Dual-Mode Type I/II Photosensitization of a Stable Mesoporous Hydrogen-Bonded Organic Framework for Antibacterial Therapy

Yi-Lun Cheng[2,3], Hui Yuan[2], Yuhang Li[2], Zi-Yu Wang[2], Rui Wang[2], Qi Yin[2,3], Xue-Ning Ren[2,5], Miao Zheng[4], Yi Zeng[1*], Tian-Fu Liu[2,3*], Zaisheng Ye[1*]*

[1]Department of Gastric Surgery, Clinical Oncology School of Fujian Medical University, Fujian Cancer Hospital, No. 420 Fu-ma Road, Fuzhou 350014, China

[2]State Key Laboratory of Structural Chemistry, Fujian Institute of Research on the Structure of Matter, Chinese Academy of Sciences, Fuzhou, Fujian, 350002, China

[3]University of the Chinese Academy of Sciences, Beijing, 100049, China

[4]Department of Clinical Laboratory, Fujian Provincial Maternity and Child Health Hospital, Affiliated Hospital of Fujian Medical University, Fuzhou, Fujian, 350001, China

[5]College of Chemistry and Materials Science, Fujian Normal University, Fuzhou, Fujian, 350007, China

Email: tfliu@fjirsm.ac.cn

Contents

Experimental Section 3

1.1 Materials 3

1.2 Characterizations 3

2.1 Preparation of N,N′-di(3′,3″,5′,5″-tetrakis(4-carboxyphenyl))-1,2,6,7-tetrachloroperylene-3,4,9,10-tetracarboxylic acid diimide 4

2.2 Powder X-ray Diffraction patterns (PXRD) of PFC-513 for stability test 6

2.3 N_2 sorption isotherm of PFC-513 for stability test and pore size and pore structure of PFC-513 8

2.4 Solvent processibitity of PFC-513 9

2.5 TEM image 11

2.6 SEM images 12

2.7 UV visible spectrum and band gap of PFC-513 13

2.8 Photoelectric properties 14

2.9 Fluorescence properties of Monomer and PFC-513[3] 15

2.10 Photochemical radical generation from PFC-513. 18

2.11 The singlet oxygen production of PFC-513 and Monomer [4] 19

2.12 The type I ROS production of PFC-513 and Monomer 20

2.13 X-ray photoelectron spectroscopy (XPS) measurements 21

2.14 CV curves measurement 22

2.15 The Fourier Transform Infrared (FT-IR) spectrum of Monomer and PFC-513 22

2.16 Water contact angle of Monomer and PFC-513 23

2.17 The electrostatic potential of PFC-513 24

2.18 The photothermal performance and photothermal conversion efficiency of PFC-513 and Monomer 24

2.19 Antibacterals of PFC-513 26

2.20 Cell biocompatibility of PFC-513 30

2.21 Biocompatibility of PFC-513[6] 31

2.22 Hemolysis test of PFC-513 33

2.23 Biostatistics of PFC-513 34

2.24 RNA sequencing (RNA-seq) analysis 41

Experimental Section

1.1 Materials

Unless otherwise specified, all reagents and solvents were purchased from commercial sources and used as received without further purification. These reagents include N, N-dimethylformamide (DMF, AR), dimethyl sulfoxide (DMSO, AR), propionic acid (AR), triethylamine (AR), methanol (MeOH, AR), ethanol (EtOH, AR), acetone (AR) and 5,5-Dimethyl-1-pyrroline N-oxide (DMPO)，indium tin oxide (ITO) glass, 1,6,7,12-Tetrachloroperylene Tetracarboxylic Acid Dianhydride, 3,5-Dibromoaniline, 4-Carboxyphenylboronic Acid, salicylic acid, Dihydrorhodamine 123 (DHR123).

1.2 Characterizations

The powder X-ray diffraction (PXRD) patterns were recorded using a Rikagu Miniflex 600 Benchtop and Rikagu Smartlab equipped with Cu Kα radiation (λ = 1.54056 Å). Prior to gas adsorption measurements, all samples were degassed at 90°C for 10 h. N_2 isotherms at 77 K were measured using an ASAP 2460 surface area and porosimetry analyzer. Data analysis was performed using ASAP 2460 (V2.01). Surface areas and experimental pore size distributions were determined according to the Brunauer–Emmett–Teller (BET) adsorption model. Fourier transform infrared (FT–IR) spectra were conducted using a VERTEX 70 series FT–IR spectrometer with ATR model. Proton nuclear magnetic resonance (^1H NMR) experiments were performed on a JOEL 400 spectrometer. Thermogravimetric analyses (TGA) were carried out under a N_2 atmosphere with a heating rate of 5°C/min using a Netzsch TGA instrument. Electron paramagnetic resonance (EPR) measurements were performed at X-band (~9.8 GHz) using a Bruker BioSpin EPR spectrometer. Scanning electron microscopy (SEM) images were collected using a JSM6700-F field emission SEM and Phenom G2 Field Emission Scanning Electron Microscope and equipped Energy Dispersive Spectroscopy (EDS) system at an acceleration voltage of 15 kV. Diffuse reflectance spectra (DRS) and solid ultraviolet–visible–near–infrared (UV–Vis–NIR) absorbance spectra were recorded at room temperature on a Shimadzu UV-2550 and UV-2600i spectrophotometer with $BaSO_4$ as blank. Steady-state photoluminescence spectra were

measured using an FS5 spectrofluorometer under ambient conditions. Fluorescence lifetimes were measured using a FLS1000 spectrofluorometer. Zeta potentials of nanoparticles in water were collected by a BI-200SM Analyzer. Static water contact angles were measured using a JC200D instrument. Powdery samples were deposited onto FTO-coated glasses substrateswith Nafion solution adhesion, forming thin films for electrochemical measurements. Cyclic voltammetry (CV) curves, Mott–Schottky plots, EIS and photocurrent were measured using a electrochemical analyzer (Zahner, Germany). X-Ray photoelectron spectroscopy (XPS) was performed on an ESCALAB 250Xi spectrometer equipped with an Al-Kα X-ray source with C 1s peak at 284.6 eV as internal standard. An 660 nm LED lamp emitting light was employed to irradiate samples for photothermal testing. A HIKMICRO H11 infrared thermal imaging camera was used to monitor the temperature. The Confocal fluorescence microscopy images were obtained by the Nikon C2.

2.1 Preparation of N,N′-di(3′,3″,5′,5″-tetrakis(4-carboxyphenyl))-1,2,6,7-tetrachloroperylene-3,4,9,10-tetracarboxylic acid diimide

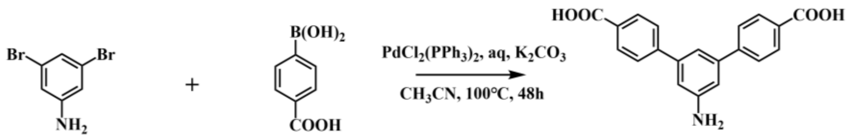

Figure S1. Synthesis route of aniline-3,5-dibenzoic acid.

250 mL Schlenk flask was charged with 3,5-dibromoaniline (2.2 g, 1 Eq, 8.8 mmol) and 4- boronobenzoic acid (3.2 g, 2.2 Eq, 19 mmol) and evacuated/backfilled with argon. Acetonitrile (40 mL) followed by K_2CO_3 (9.7 g, 35 mL, 8.0 Eq, 70 mmol) were added to the mixture and then solution was bubbled with N_2 for 1h. To this $Pd(PPh_3)Cl_2$ (0.37 g, 6 mol%, 0.53 mmol) was added, flask was sealed and placed in preheated to 100°C oil bath for 48 h. Cooled to room temperature, diluted with water (200 ml), filtered and washed thoroughly with water. The filtrate was acidified to pH ~3 with 1M HCl, the precipitate was collected by filtration, washed with water, followed by hexane, dried briefly on air, then at high vacuum at 50°C overnight to give aniline-3,5 dibenzoic acid (2.80 g, 96%) as off-white powder.[1]

^{1}H NMR (600 MHz, DMSO-d6) δ 12.92 (s, 2H), 8.02 (d, J = 8.2 Hz, 4H), 7.79 (d, J = 8.2 Hz, 4H), 7.20 (s, 1H), 6.99 (s, 2H), 5.67 (s, 2H) ppm.

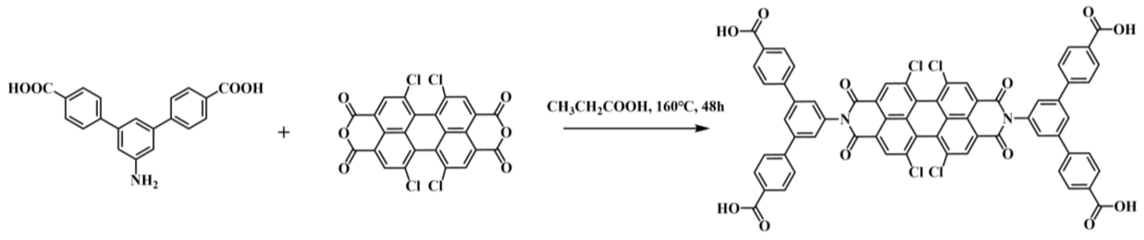

Figure S2. Synthesis route of N,N′-di(3′,3″,5′,5″-tetrakis(4-carboxyphenyl))-1,2,6,7-tetrachloroperylene-3,4,9,10-tetracarboxylic acid diimide.

N,N′-di(3′,3″,5′,5″-tetrakis(4-carboxyphenyl))-1,2,6,7-tetrachloroperylene-3,4,9,10-tetracarboxylic acid diimide was synthesized according to the literature with improvement. A suspension of 1,6,7,8-tetrachloroperylene-3,4,9,10-tetracarboxylic acid dianhydride (0.53 g, 1mmol) and propionic acid (25 mL) were loaded into a 100 mL round-bottom flask and stirred for 5 minutes. To this solution, aniline-3,5-dibenzoic acid (1 g, 3 mmol) was added and the solution was stirred under reflux at 150 °C for 24 h. After the reaction was cooled to room temperature, water was added to precipitate the product. The solid was collected by filtration and washed with ethanol and dried in a vacuum to yield a red solid (1 g, 86 %). [2]

^{1}H NMR (600 MHz, DMSO-d6) δ 13.06 (s, 4H), 8.65 (s, 4H), 8.23 (s, 2H), 8.10 - 8.06 (m, 8H), 8.00 - 7.94 (m, 12H).

It is mentioned that PFC-513 was obtained by adjusting the stirring rate of the magnetic stirrer during this step of the organic synthesis and PFC-513 was subsequently purified by washing with ethanol.

2.2 Powder X-ray Diffraction patterns (PXRD) of PFC-513 for stability test

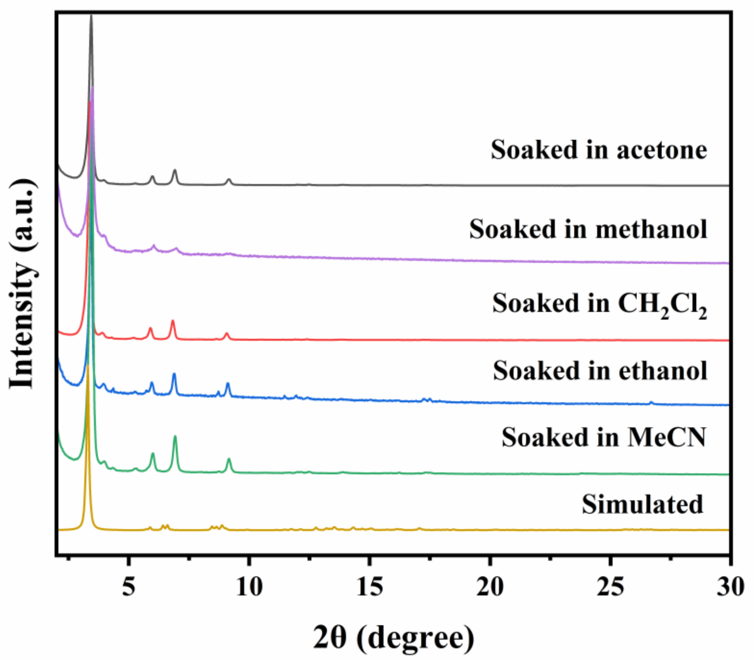

Figure S3. The PXRD patterns of PFC-513 after being treated with different solution for a weak.

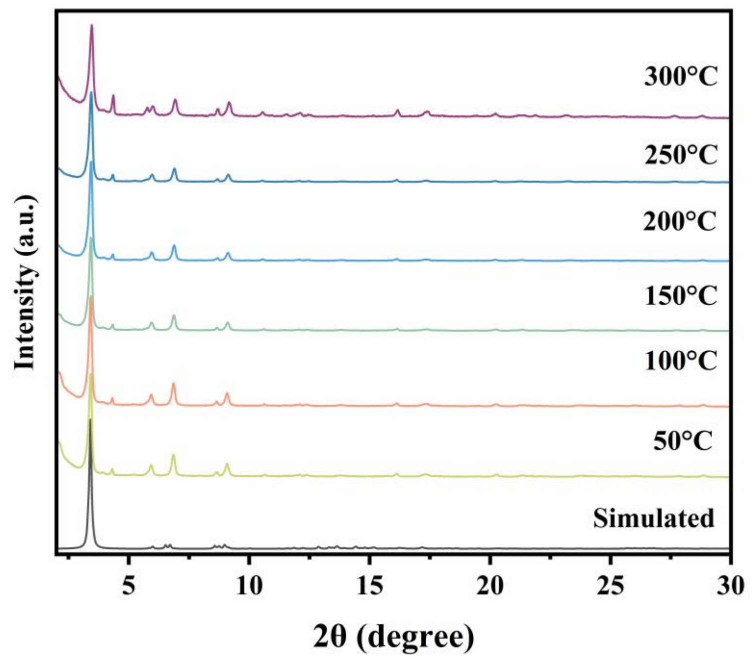

Figure S4. Variable temperature PXRD patterns of PFC-513.

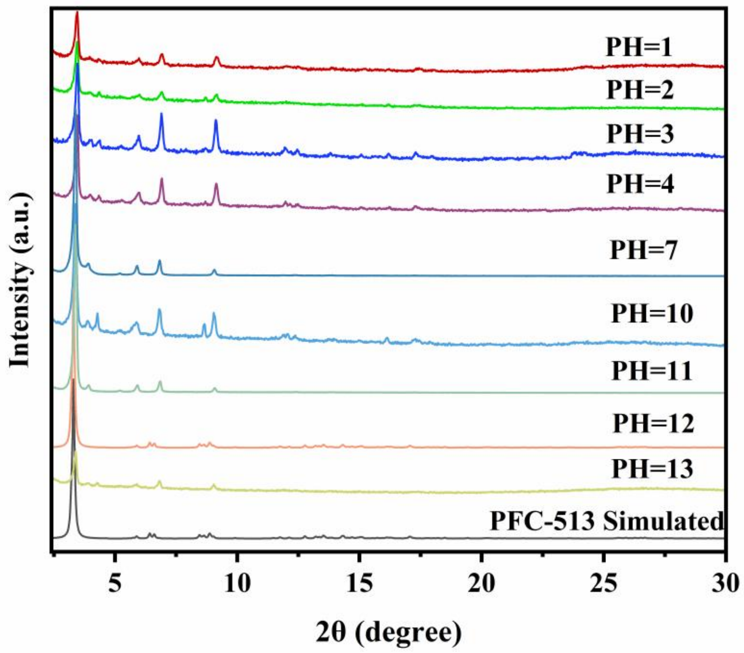

Figure S5. The PXRD patterns of PFC-513 after being treated with different pH aqueous solution for a month.

2.3 N_2 sorption isotherm of PFC-513 for stability test and pore size and pore structure of PFC-513

The N_2 isotherms were measured using ASAP 2460 from Micromeritics Co. Ltd. The as-prepared sample was washed with CH_2Cl_2 3 times. Then the sample was allowed to soak in CH_2Cl_2 for 96 h with the supernatant being replaced by fresh CH_2Cl_2 several times during the process to exchange and remove nonvolatile solvates (H_2O and propionic acid). After removal of CH_2Cl_2 by centrifugation, the samples were activated under vacuum at room temperature, and then dried again in the "outgas" function of instruments at 120 °C for 10 hours for PFC-513 prior to gas adsorption. The N_2 isotherm measurements were performed at 77 K and a pressure of 1 bar. We utilized NLDFT to calculate the pore size of PFC-513.

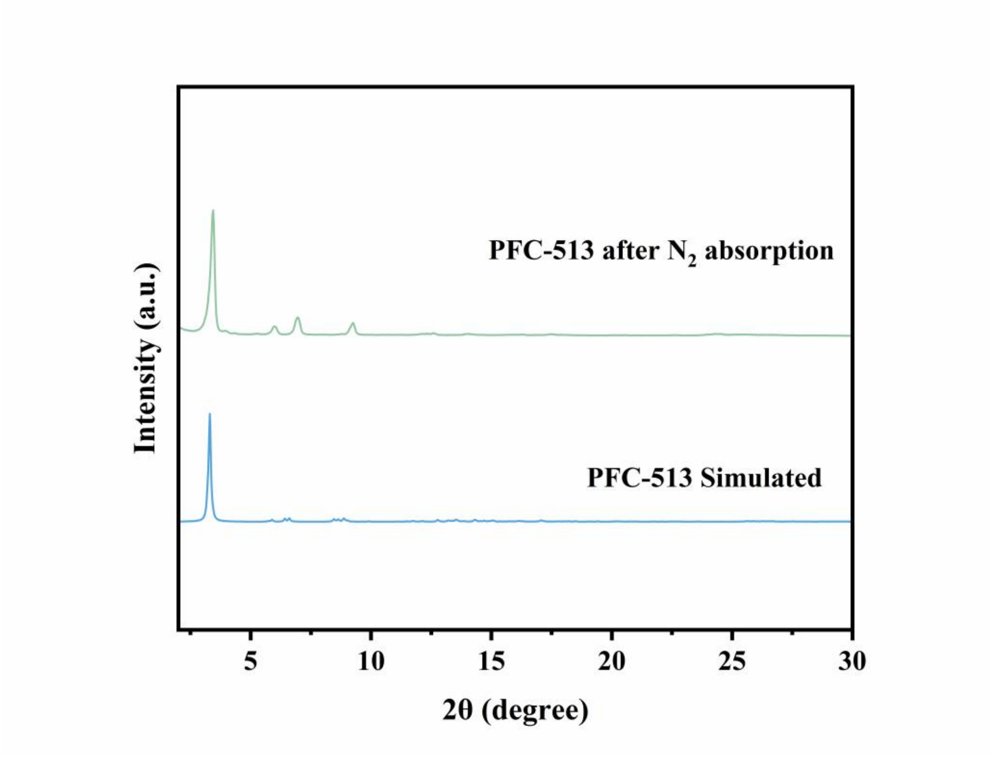

Figure S6. PXRD pattern of PFC-513 after the N_2 sorption measurement.

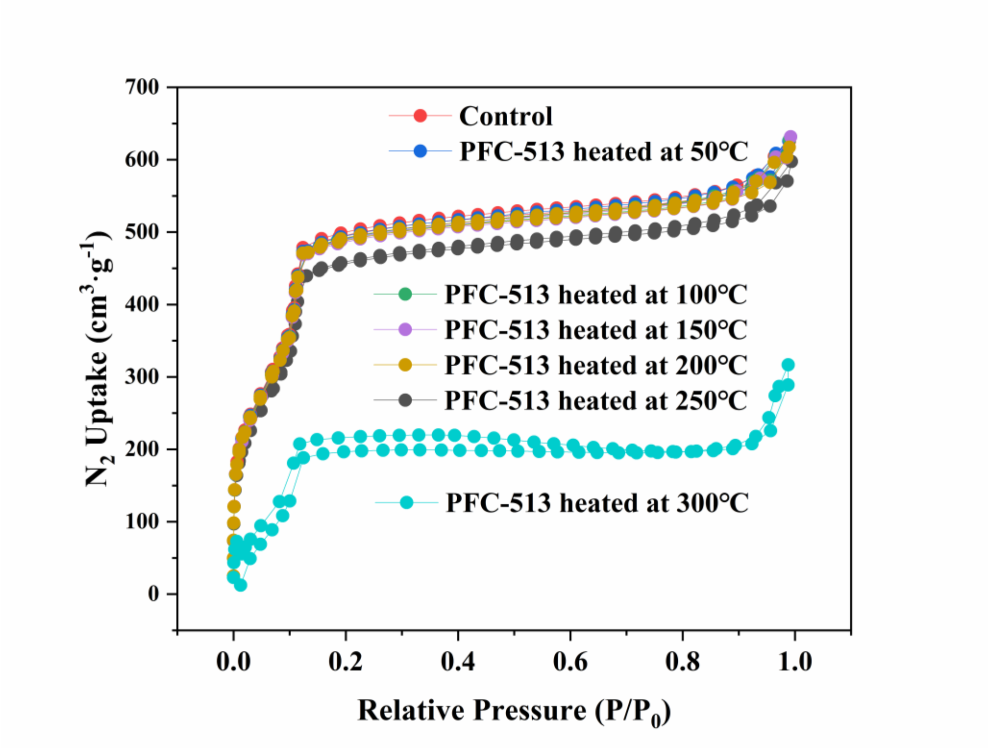

Figure S7. The N_2 sorption of PFC-513 after heated different temperature for 6 hours.

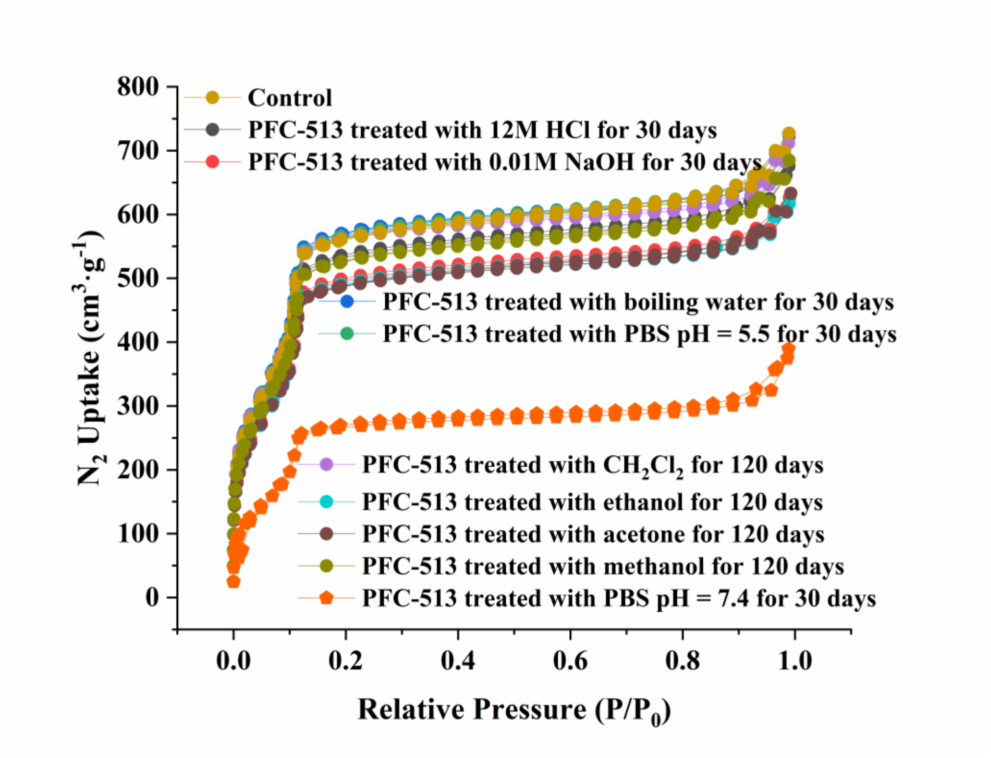

Figure S8. The N_2 sorption of PFC-513 after heated different solution.

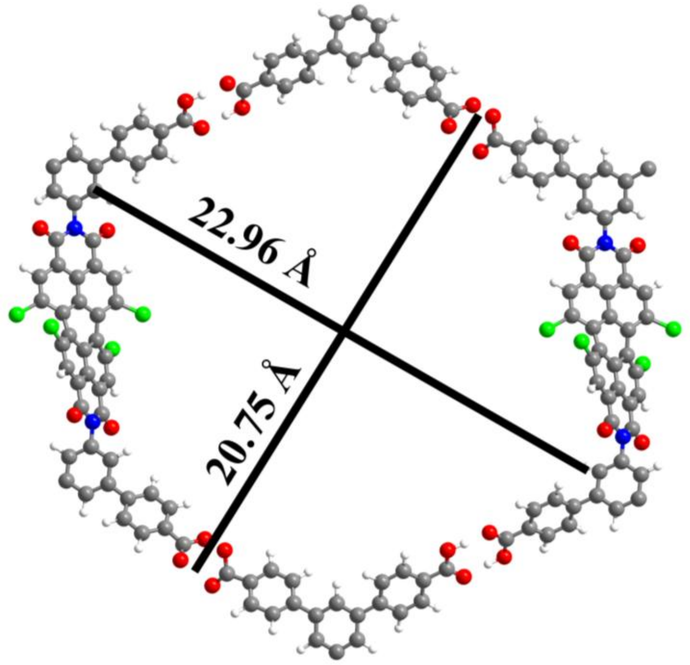

Figure S9. The pore size and pore structure of PFC-513.

2.4 Solvent processibility of PFC-513

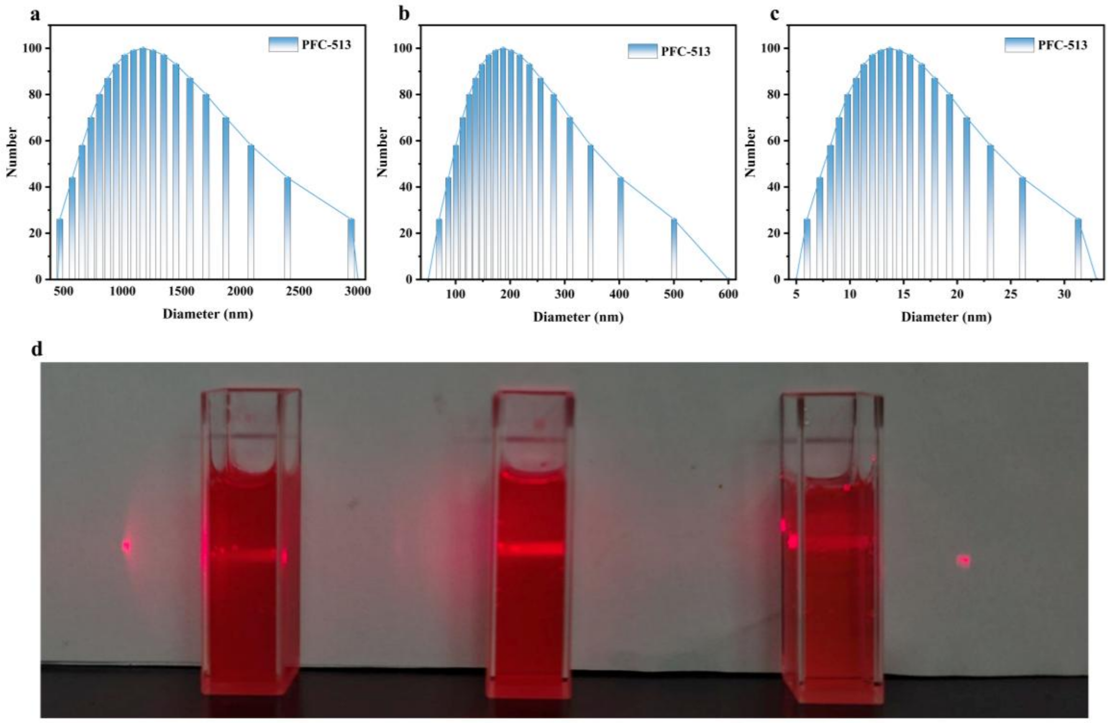

Figure S10. Concentration-dependent particle size distribution of PFC-513. DLS analysis reveals the evolution of hydrodynamic diameter with decreasing concentration: a) 30, b) 5, and c) 0.5 mg/mL. d) Tyndall effect of PFC-513 aqueous dispersions with graded particle sizes upon irradiation with a 650 nm laser, showing from left to right are structures with diameters of 1500 nm, 300 nm, and 20 nm.

The PFC-513@PAAMs composite hydrogel was prepared by first dissolving polyvinylpyrrolidone (PVP, 0.2 g), polyacrylamide (PAAM, 0.8 g), polyethylene glycol (PEG, 1.0 g), and agar (2.0 g) in deionized water (95 mL). This mixture was then autoclaved for 2 h to yield a homogeneous viscous liquid, which was cooled to 50 °C before the addition of pre-synthesized PFC-513. After stirring for 2 minutes to achieve homogeneity, the mixture was transferred to a sterile circular polystyrene Petri dish and cooled to room temperature to form the final hydrogel.

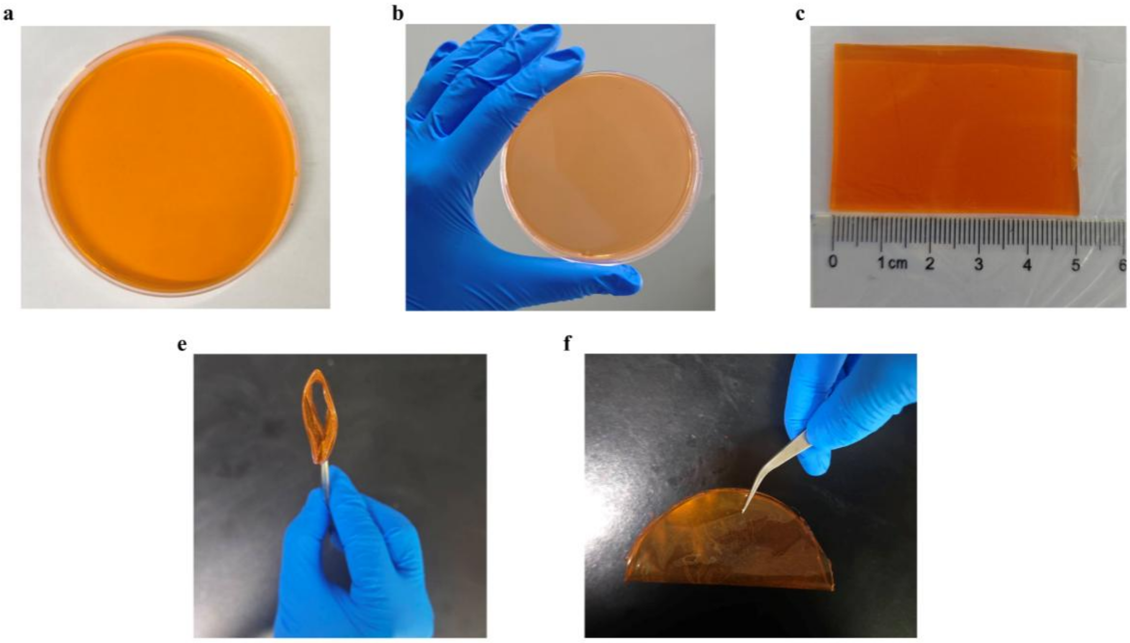

Figure S11. The PFC-513@PAAMs hybrid matrix hydrogel membrane fabricated via solvent processibility treatment.

2.5 TEM image

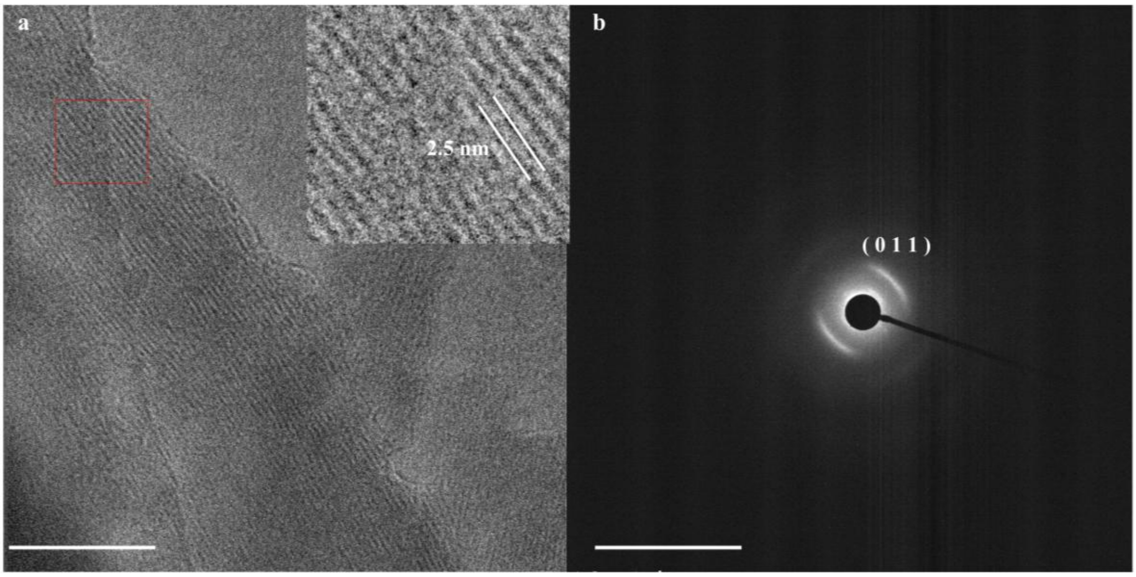

Figure S12. a) HRTEM images of PFC-513 (Inset: lattice fringe images of PFC-513). b) Selected-area electron diffraction (SAED) pattern of PFC-513 (scale bar: 50 nm).

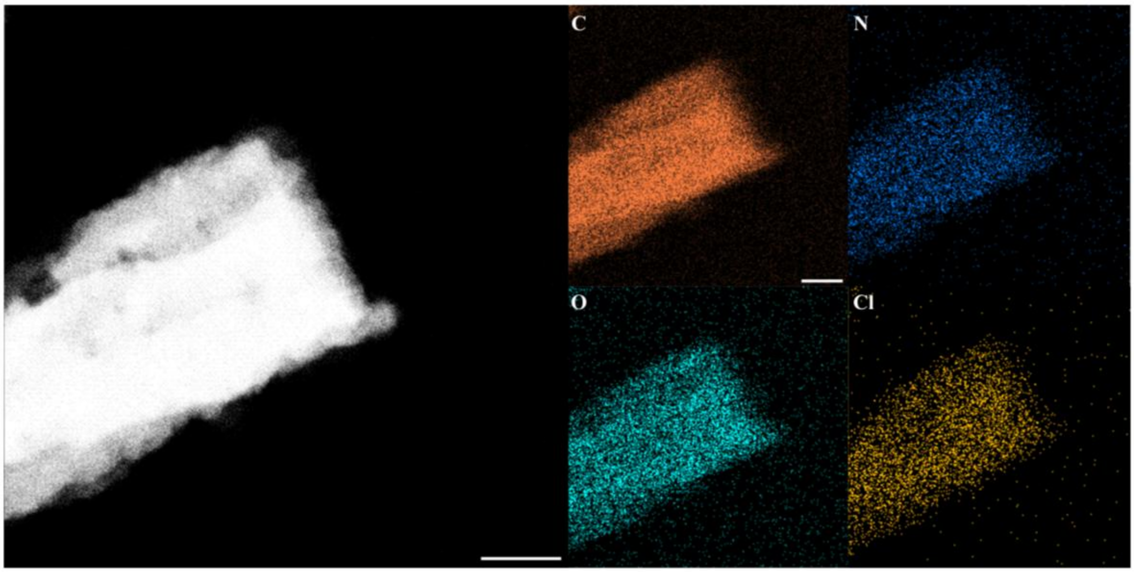

Figure S13. HRTEM and EDX mapping images of PFC-513 (scale bar: 50 nm).

2.6 SEM images

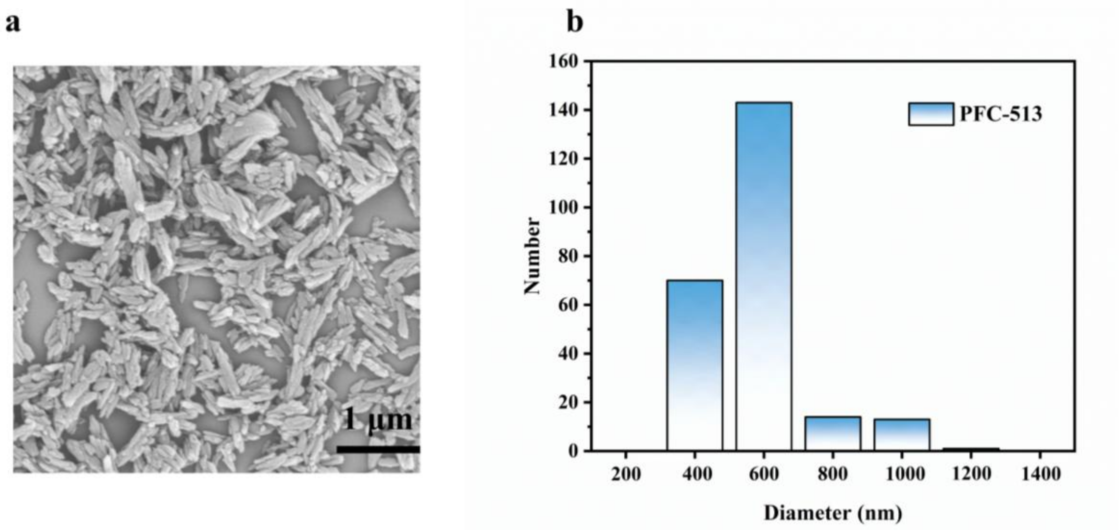

Figure S14. SEM images and particle size distribution of PFC-513.

2.7 UV visible spectrum and band gap of PFC-513

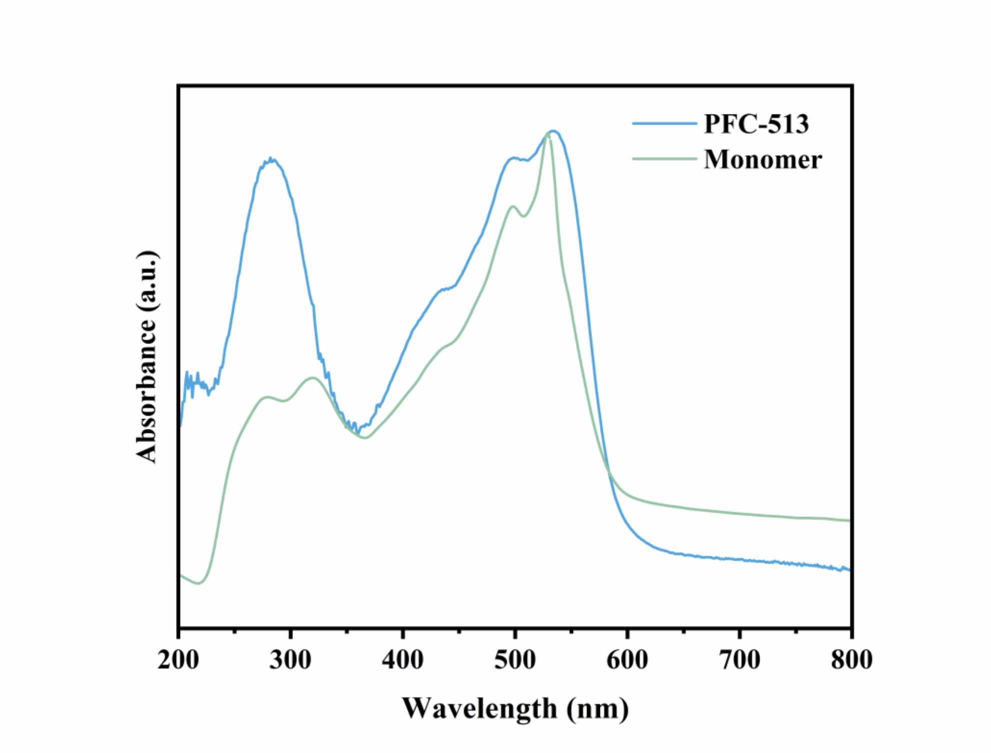

Figure S15. The UV visible spectrum of PFC-513 and Monomer.

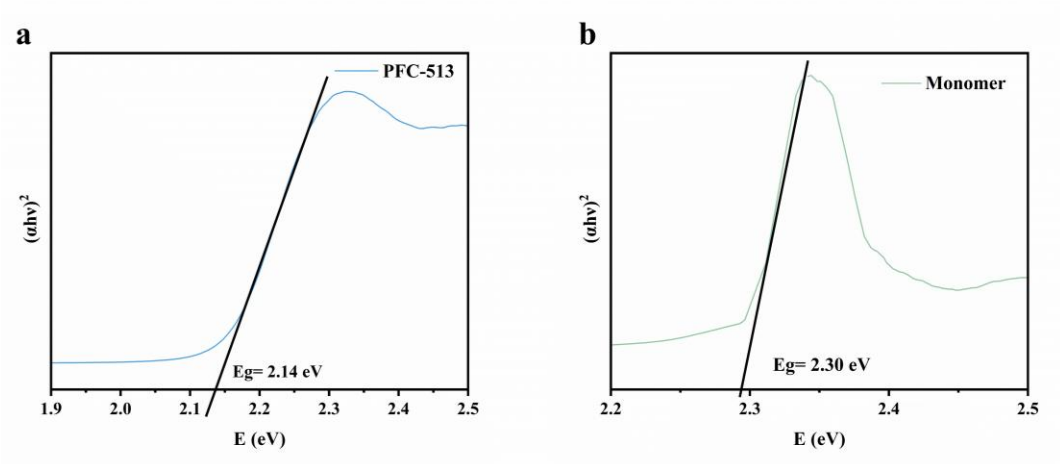

Figure S16. The band gap of PFC-513 and Monomer.

2.8 Photoelectric properties

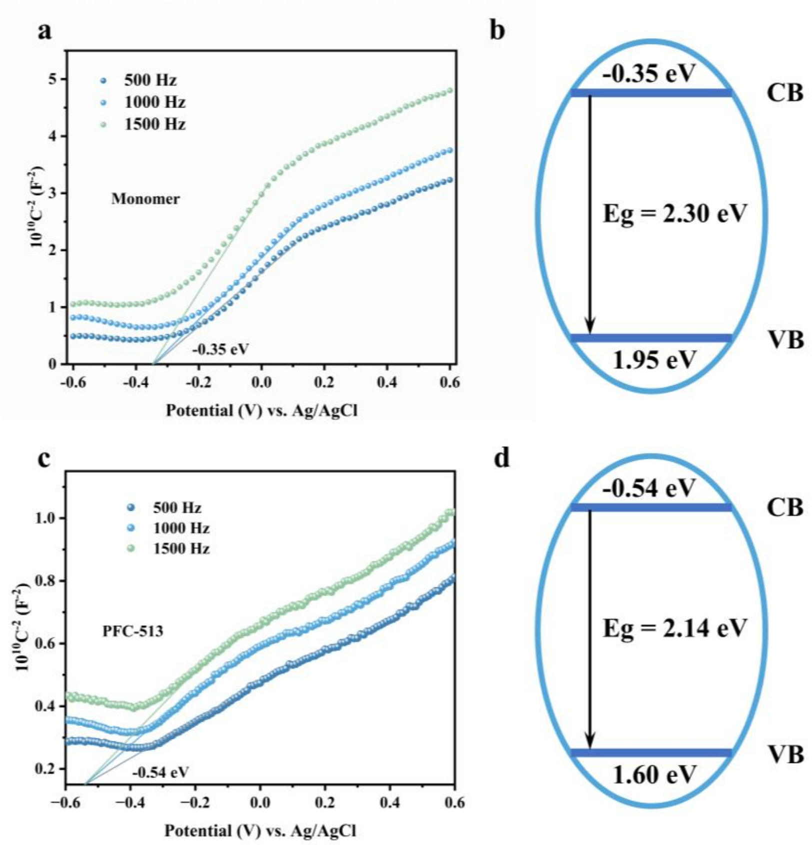

Figure S17. Mott Schottky plots of Monomer and PFC-513.

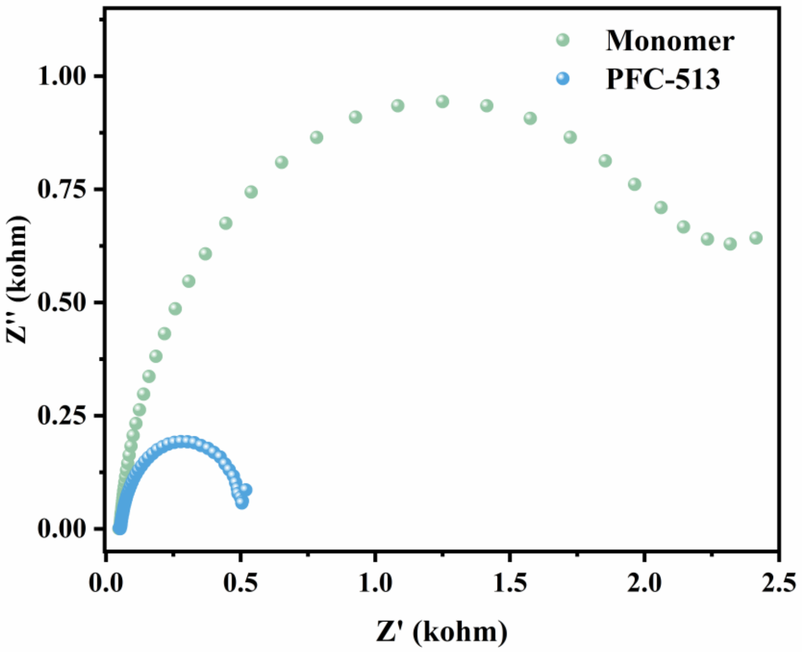

Figure S18. The electrochemical impedance spectroscopy of Monomer and PFC-513.

2.9 Fluorescence properties of Monomer and PFC-513[3]

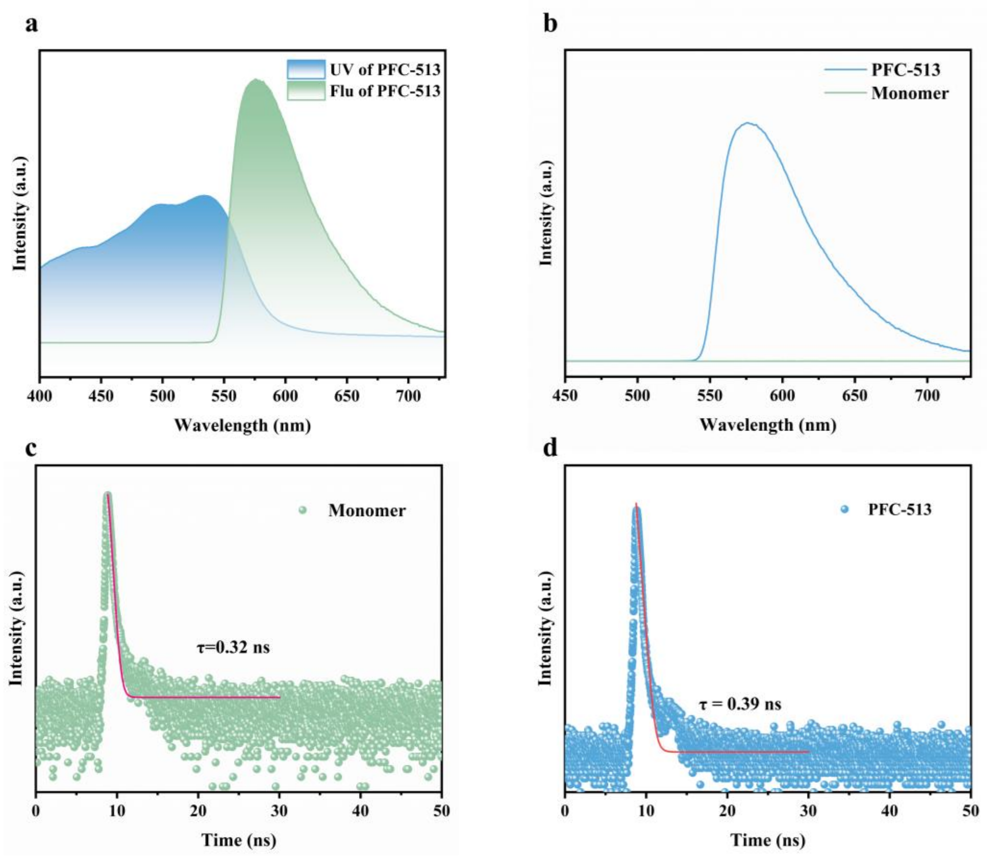

Figure S19. a) The solid-state fluorescence spectrum and UV absorption spectrum of PFC-513, b) Fluorescence spectra of PFC-513 and Monomer. The fluorescence lifetime of c) Monomer and d) PFC-513

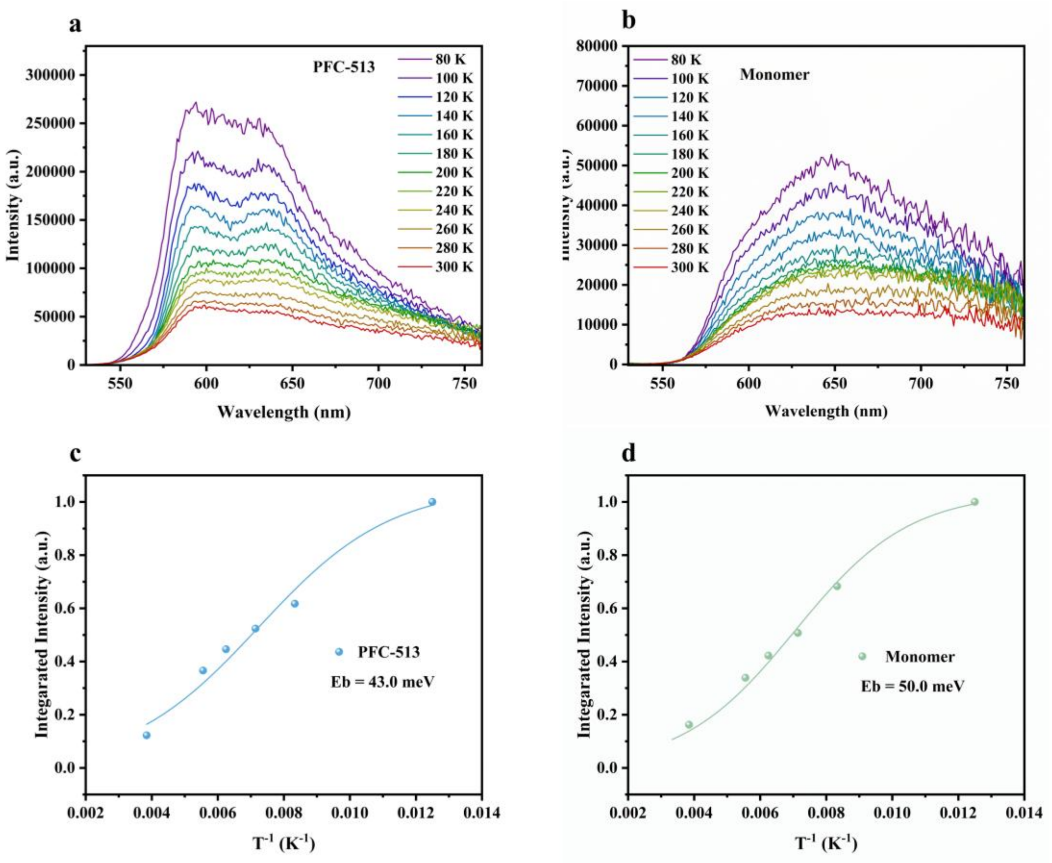

Figure S20. Temperature dependent fluorescence spectroscopy of a) PFC-513 and b) Monomer. The fitted exciton binding energy of c) PFC-513 and d) Monomer.

The fluorescence decays are fitted by a three-exponential function:

Equation S1:

$$y=y_0+A_1\,exp\,(-\frac{t}{\tau_1})+A_2\,exp\,(-\frac{t}{\tau_2})$$

Where A and τ are amplitude and time constant, respectively. The average photoluminescence lifetimes were calculated as follows:

Equation S2:

$$\tau_{ave.}=\frac{A_1\tau_1^2+A_2\tau_2^2}{A_1\tau_1+A_2\tau_2}$$

Table S1. Parameters for fitting the kinetic curves of ultrafast TA spectra

Sample	A_1	τ_1 (ns)	A_2	τ_2 (ns)	τ_{ave} (ns)
**Monomer**	1.63E12	0.32	-1.76	0.12	0.32
**PFC-513**	7.20E9	0.38	7.12E9	0.38	0.39

In a typical ultrafast TA measurement, the light from pulsed laser was split into two parts, one light (pump) promotes the samples from ground-state to excited state, and the other light (probe) is for measuring the absorbance of molecules in ground state. A differential TA spectrum (△A) is deduced by subtracting the absorption spectrum of the pumped sample to the absorption spectrum of the non-pumped sample. The ground-state bleach (GSB) signal reflects the population of molecules in the ground-state. As a portion of molecules were promoted to excited state by the pump light, the number of molecules in the ground-state would decrease. Therefore, the ground state absorption in the excited sample is less than that in the non-excited sample, generating a negative signal in △A spectrum.

The TA decay kinetics probing at 549 nm are fitted by a three-exponential function:

Equation S3:

$$y=y_0+B_1 \exp(-\frac{t}{\tau_1})+B_2 \exp(-\frac{t}{\tau_2})+B_3 \exp(-\frac{t}{\tau_3})$$

Where B and τ are the amplitude and the time constant, respectively. The TA average lifetimes were calculated as follows:

Equation S4:

$$\tau_{ave.}=\frac{B_1\tau_1^2+B_2\tau_2^2+B_3\tau_3^2}{B_1\tau_1+B_2\tau_2+B_3\tau_3}$$

Table S2. Parameters for fitting the kinetic curves of ultrafast TA spectra

Sample	B_1	τ_1 (ns)	B_2	τ_2 (ps)	A_3	B_3 (ns)	τ_{ave} (ns)
**Monomer**	0	0.016	0	0.00012	0	0.00012	0
**PFC-513**	2.38E-4	9.428	4.19E-4	2.546	3.08E-4	84.61	0.08125

2.10 Photochemical radical generation from PFC-513.

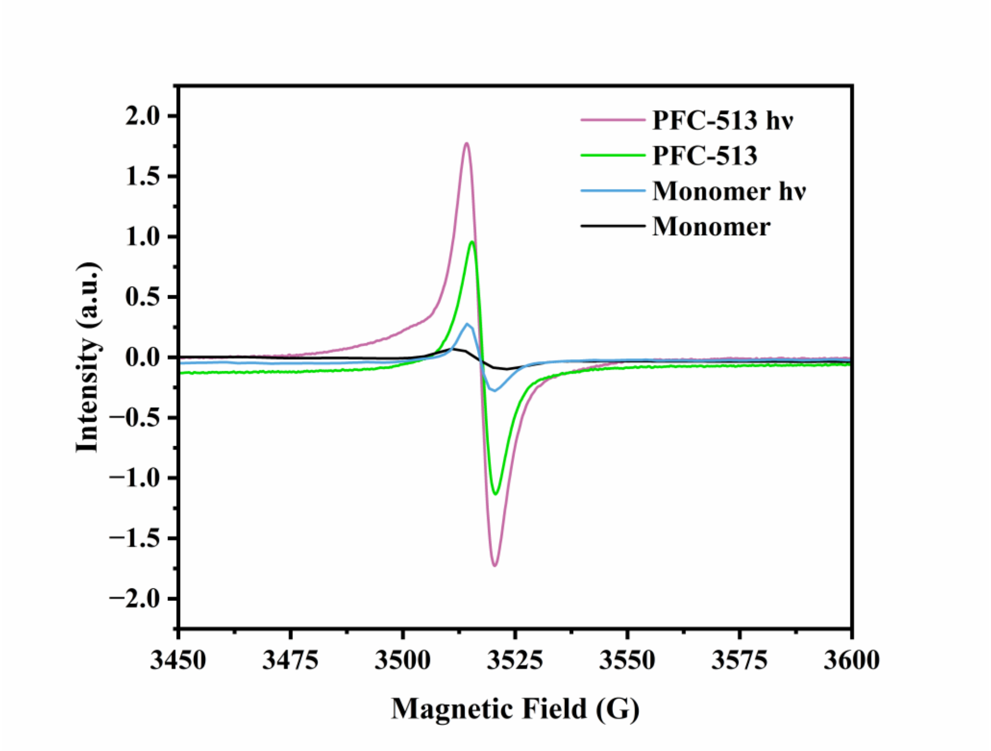

Figure S21. EPR spectra of PFC-513 and Monomer before and after irradiation.

2.11 The singlet oxygen production of PFC-513 and Monomer [4]

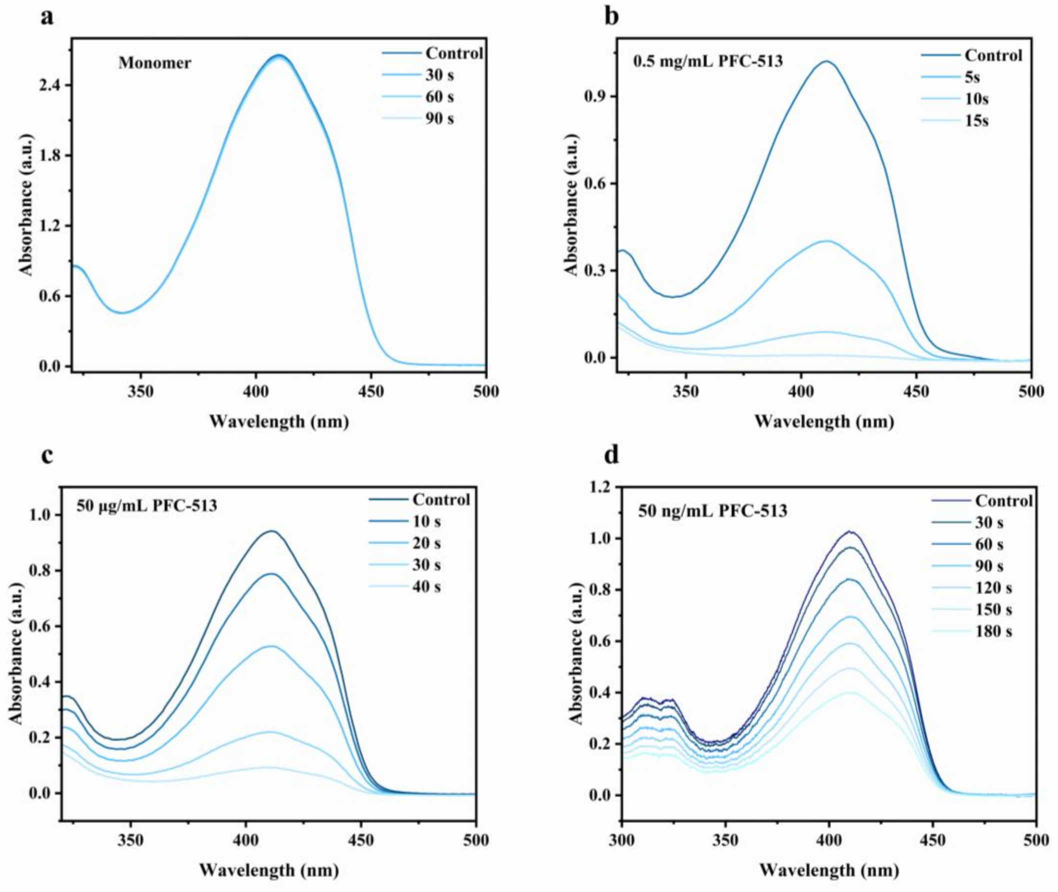

Figure S22. Time-dependent UV-Vis absorption of DPBF in the presence of a) 0.5 mg/mL Monomer b) 0.5 mg/mL PFC-513 c) 50 μg/mL PFC-513 d) 50 ng/mL under laser irradiation with 525 nm.

The singlet oxygen ($^1O_2$) rate constant of samples was determined by the **Equation S5**,

Equation S5: $ln\,(A/A_0)=-kt$

Where A and A_0 represent the absorbance of DPBF at t and t_0, respectively.

The singlet oxygen ($^1O_2$) quantum yield of PFC-513 was determined by the **Equation S6**.

$$\textbf{Equation S6:}\ \Phi_{\triangle} = \Phi_{\triangle(\mathrm{st})} * \frac{k}{k_{\mathrm{st}}} * \frac{A_{\mathrm{st}}}{A}$$

where k is the rate constant of the degradation of DPBF. A is the absorption correction factor. The subscript of st represents the standard sample (RB).

Table S3. Parameters and values in the photothermal conversion rate equations

Samples	k	$A_{(475nm)}$	ΦΔ
**Monomer**	≈ 0	2.5	≈ 0
**PFC-513**	0.07	1.71	0.98
**RB**	0.007	0.31	0.54

2.12 The type I ROS production of PFC-513 and Monomer

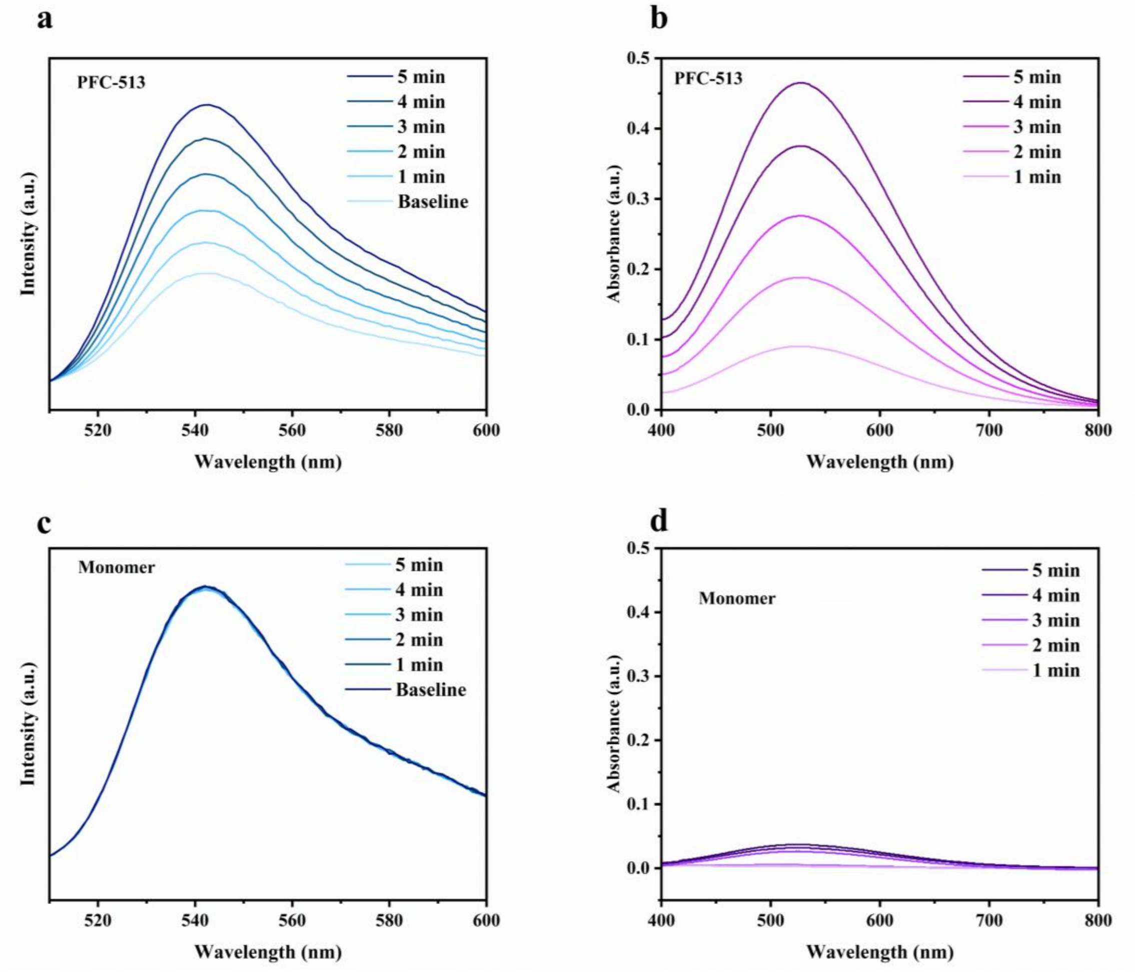

Figure S23. Time-dependent fluorescence of DHR123 in the presence of a) 0.5 mg/mL PFC-513 c) 0.5 mg/mL Monomer under laser irradiation with Xe lamp. Time-dependent UV-Vis absorption of salicylic acid in the presence of b) 0.5 mg/mL PFC-513 d) 50 mg/mL Monomer under laser irradiation with Xe lamp.

2.13 X-ray photoelectron spectroscopy (XPS) measurements

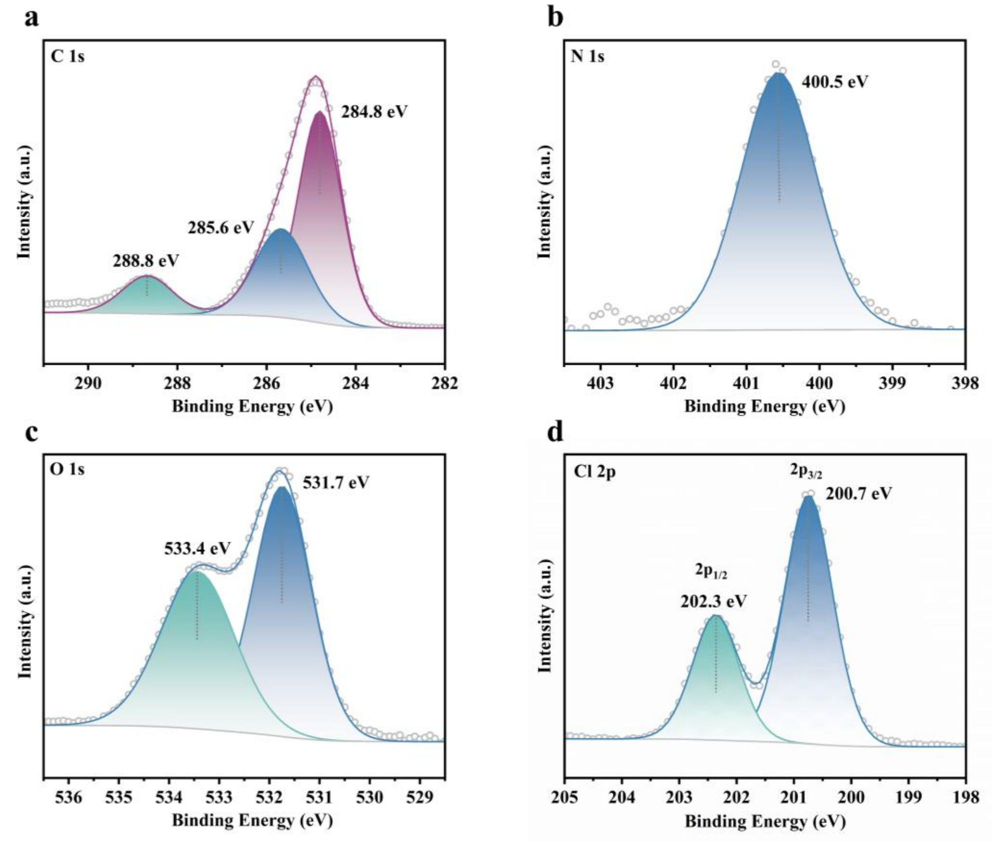

Figure S24. XPS analyses of PFC-513.

2.14 CV curves measurement

A standard three-electrode cell was used with Ag/AgCl (saturated KCl solution) as reference electrode and Pt electrode as counter electrode. 5 mg sample was dispersed in the solution consisting of 450 μL ethanol and 50 μL 5% Nafion by ultrasonication for 30 min. Then, 100 μL (20 μL each time) of the suspension was pipetted in a 1×1 cm^2 area on an indium tin oxide (ITO) glass and dried naturally at room temperature to serve as the working electrode. All the experiments were conducted in 0.2 M Na_2SO_4 electrolyte at room temperature.

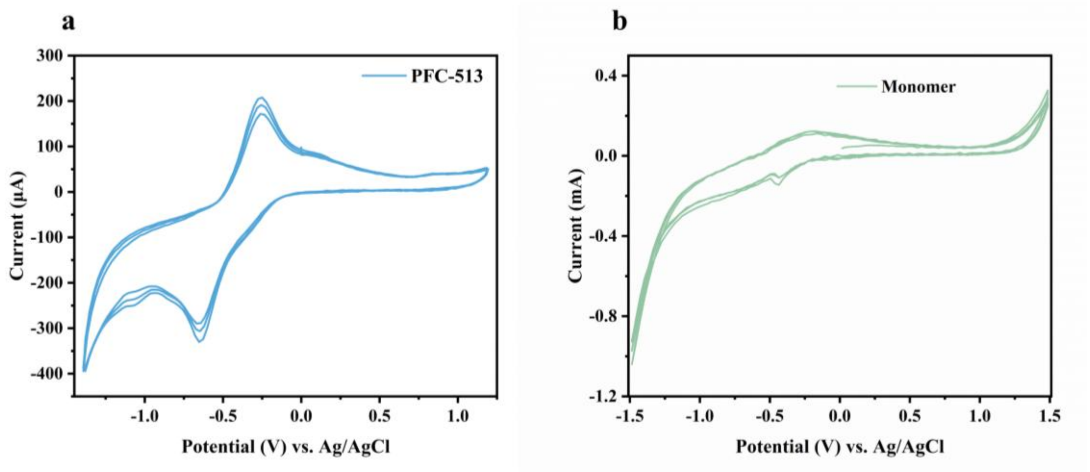

Figure S25. Cyclic voltammetry curves for a) PFC-513 and b) Monomer, 3-cycle scans at a rate of 50 mV/s.

2.15 The Fourier Transform Infrared (FT-IR) spectrum of Monomer and PFC-513.

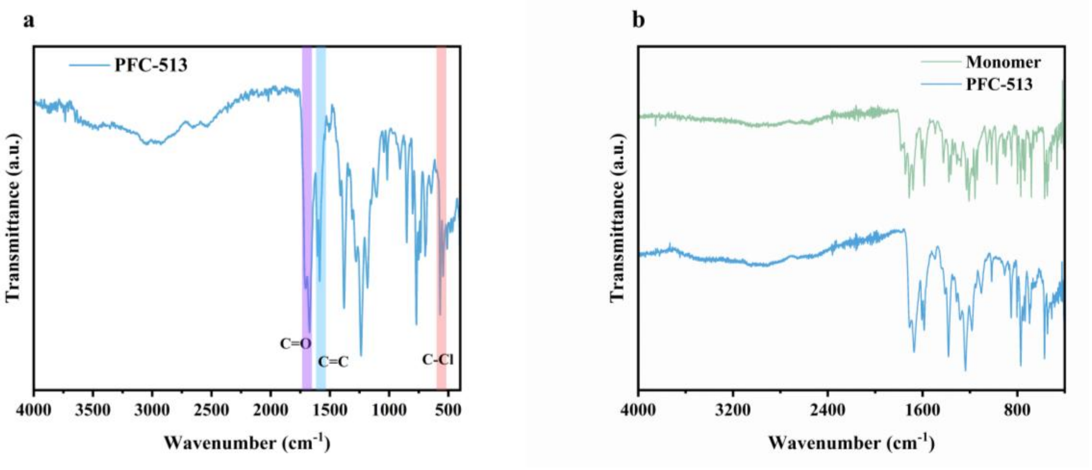

Figure S26. a) The FT-IR spectrum of PFC-513 and special bond. b) The FT-IR spectrum of PFC-513 and Monomer.

2.16 Water contact angle of Monomer and PFC-513

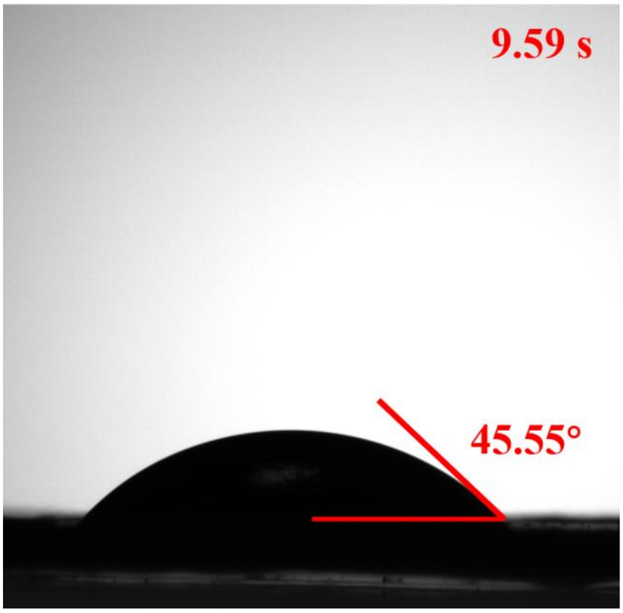

Figure S27. Water contact angle of Monomer.

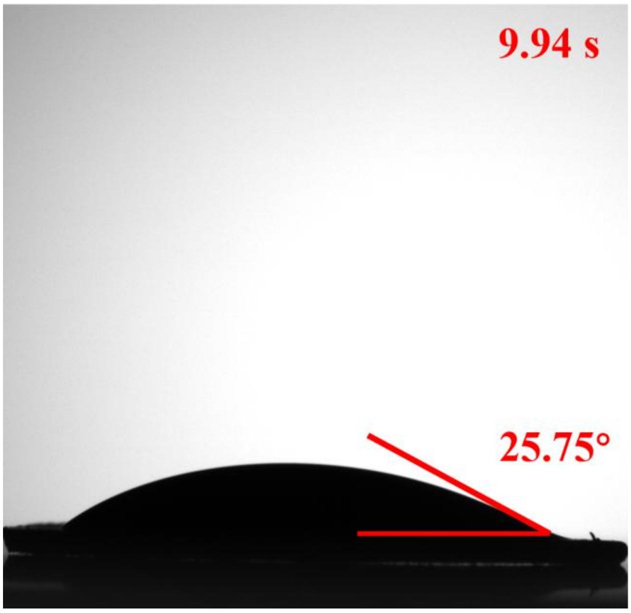

Figure S28. Water contact angle of PFC-513.

2.17 The electrostatic potential of PFC-513

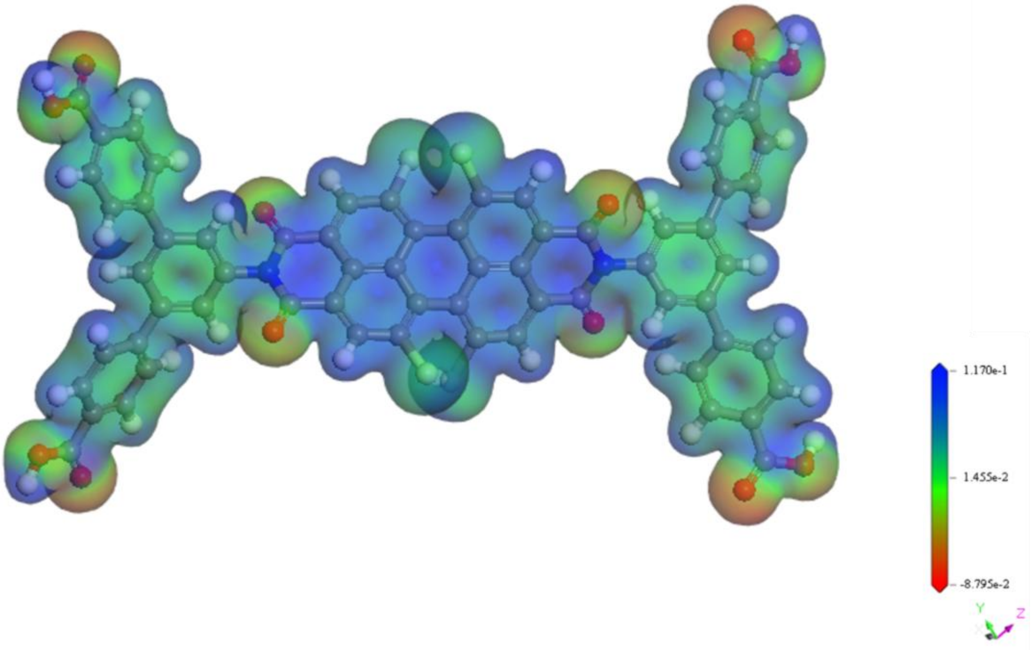

Figure S29. The electrostatic potential diagram of PFC-513.

2.18 The photothermal performance and photothermal conversion efficiency of PFC-513 and Monomer

The photothermal conversion efficiencies (PTCE, η) of PFC-513 and Monomer were calculated according to previously described methods[5], and was defined as

$$\textbf{Equation S7:}\ \boldsymbol{\eta} = \frac{\mathbf{hs}(\Delta\mathbf{T}_{\mathbf{material}} - \Delta\mathbf{T}_{\mathbf{water}})}{\mathbf{I}(\mathbf{1} - \mathbf{10}^{-\mathbf{A}})}$$

Herein, I is the laser power; A is the absorbance of aqueous suspension at the tested wavelength; $\Delta T_{material}$ and ΔT_{water} represent the temperature variation of the test sample and water, respectively; h is the heat transfer coefficient and s is the surface area of the container, which are determined from the following equation:

$$\textbf{Equation S8:}\ \mathbf{hs} = \frac{\mathbf{mc}}{\boldsymbol{\tau}_{\mathbf{s}}}$$

where m is the mass of the solution, which approximates to 2 g; c is the specific heat capacity of the solvent (4.2 J g^{-1} $°C^{-1}$ for water); τ_s is a time constant, which can be determined in cooling period from the following equation:

$$\textbf{Equation S9:}\ t = -\tau_s \ln(\theta)$$

where θ is a time dependent dimensionless parameter, known as the driving force temperature and defined as follows:

$$\textbf{Equation S10}: \theta = \frac{T - T_{surr}}{T_{max} - T_{surr}}$$

T is the actual temperature of the sample; T_{sur} is ambient temperature; T_{max} is the highest temperature of the sample.

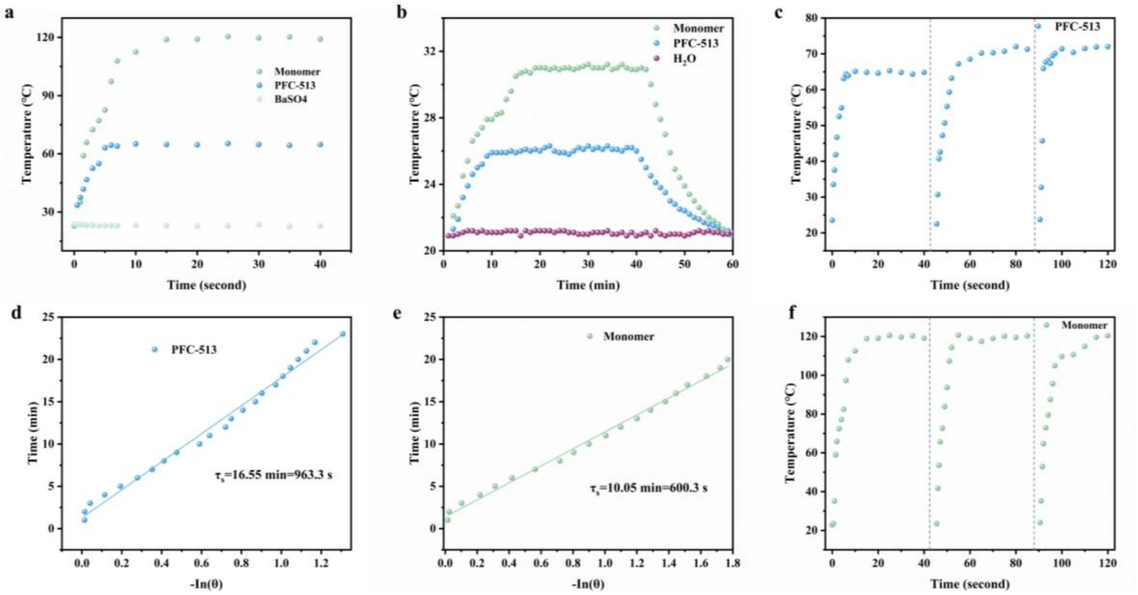

Figure S30. a) The solid-state photothermal performance of PFC-513 and Monomer. b) Photothermal conversion curves of Monomer and PFC-513 suspended in water under irradiation (660 nm, 1.15 W/cm^2) for 15 minutes and then switch the laser off; The plot of time versus -ln(*θ*) from the data recorded during the cooling period of the experiment for d) PFC-513 and e) Monomer. Photothermal cycling test of c) PFC-513 and f) Monomer. Temperature variation profiles over four cycles under 808 nm laser irradiation (1.5 W cm^{-2}).

Table S4. The parameters in the photothermal conversion rate formula

Sample	$\Delta T_{material}$-ΔT_{H_2O} (°C)	Absorbance	τ_s	hs	η (%)
**Monomer**	11.2	0.05	600.3	0.20	8.80
**PFC-513**	6.3	0.35	963.3	0.01	3.08

2.19 Antibacterials of PFC-513

In this study, E. coli and MRSA were used to evaluate the antibacterial ability of PFC-513. The optical density (OD) values of bacterial suspensions were determined at 600 nm using a microplate reader for colony counting. The bactericidal effect of PFC-513 was first assessed through a OD value assay. Bacterial suspensions (400 μL, 1 × 10^8 CFU/mL) were added to 24-well culture plates and co-cultured with equal volumes of different groups (PBS, 0.5 mg/mL PFC-513, Monomer) at 37 °C for 4 h. The bacterial suspensions were then collected and centrifuged. The treated bacterial suspensions were incubated in LB medium at 37 °C for 12 h. Afterward, the bacterial suspensions were collected and transferred to 96-well culture plates for OD measurement. The OD value of PBS solution was labeled as N_0, the OD value of the PBS-treated bacterial group was labeled as N_1, and the OD value of the test bacterial group was labeled as N_t. The bacterial killing ratio was calculated using the formula: bacterial killing ratio (%) = $[1 – (N_t – N_0)/ (N_1 – N_0)] \times 100\%$. Figure 4a and 4b present statistical results from three independent experiments (mean ± SD), whereas Figure S31 shows representative images from one experimental batch.

For colony formation assay, bacterial suspension (100 uL, 1×10^5 CFU/mL) was spread onto LB agar plates and incubated at 37 °C for 12 h to form visible colonies. Photographs of the LB plates were taken to observe and evaluate the antibacterial effect. The live-dead bacteria staining assay was used to assess bacterial membrane integrity and viability. Following co-culture with different experimental groups, the bacterial suspensions were stained using a live/dead bacterial staining kit. Specifically, 1 mL of bacterial suspension was incubated with DMAO (1.5 μL) and PI (1.5 μL) in the dark for 15 min at room temperature. The stained suspensions were then washed twice with 0.8% NaCl solution, applied to glass slides, and fluorescence images were captured using CLSM.

SEM analysis revealed distinct morphological changes in bacterial membranes among the different experimental groups. Briefly, bacterial suspensions were fixed with 2.5% glutaraldehyde for 2 h and then dispersed in deionized (DI) water. A 10 μL aliquot of the sample was deposited onto a silicon wafer, allowed to air dry at room temperature,

and subsequently coated with a thin layer of gold. At least three representative images were captured for each sample during SEM analysis.

TEM micrographs revealing the morphology of bacterial membranes under different experimental conditions. Briefly, bacterial suspensions were fixed with 2.5% glutaraldehyde for 2 h and then dispersed in deionized (DI) water. A 10 μL aliquot of the sample was deposited on a TEM grid and air-dried at room temperature. At least three representative images were captured for each sample during TEM analysis.

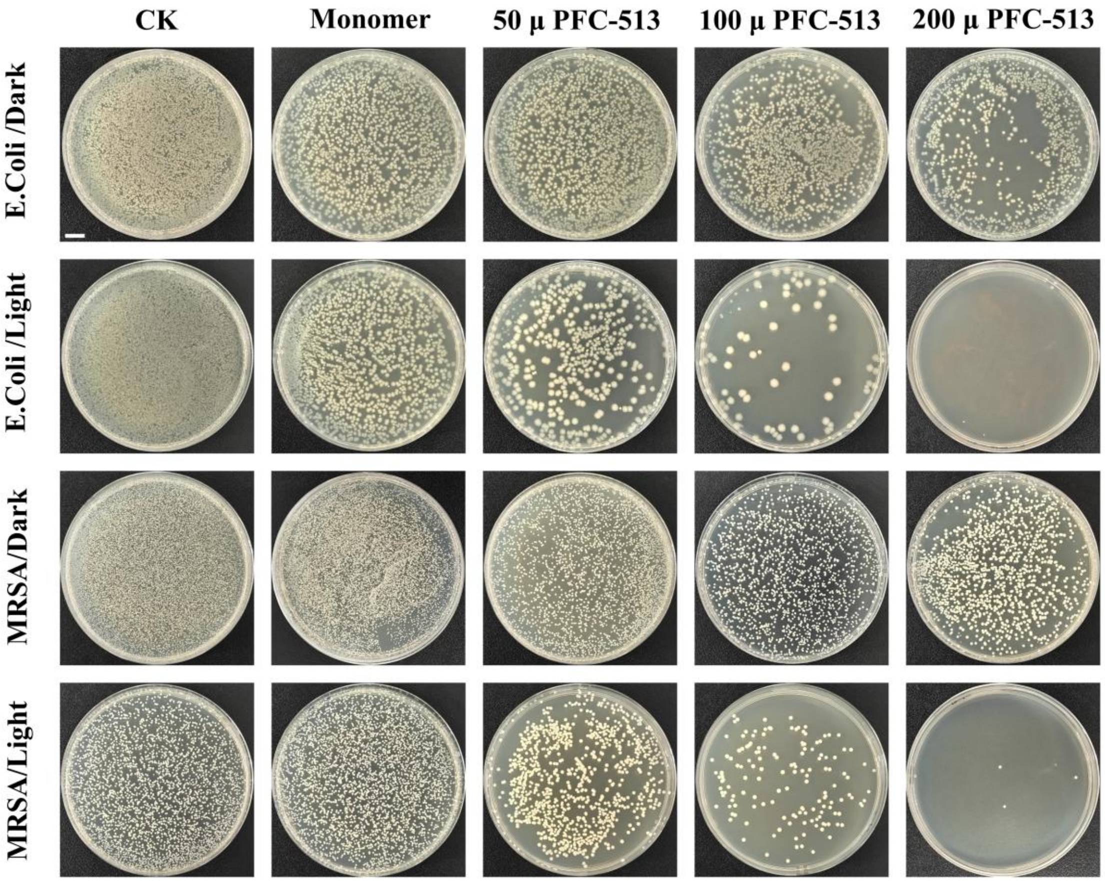

Figure S31. Experiment spread plate images of the *MRSA* and *E. coli* bacterial colonies in different PFC-513 group after three cycles of antibacterial tests (Scale bar: 1 mm)

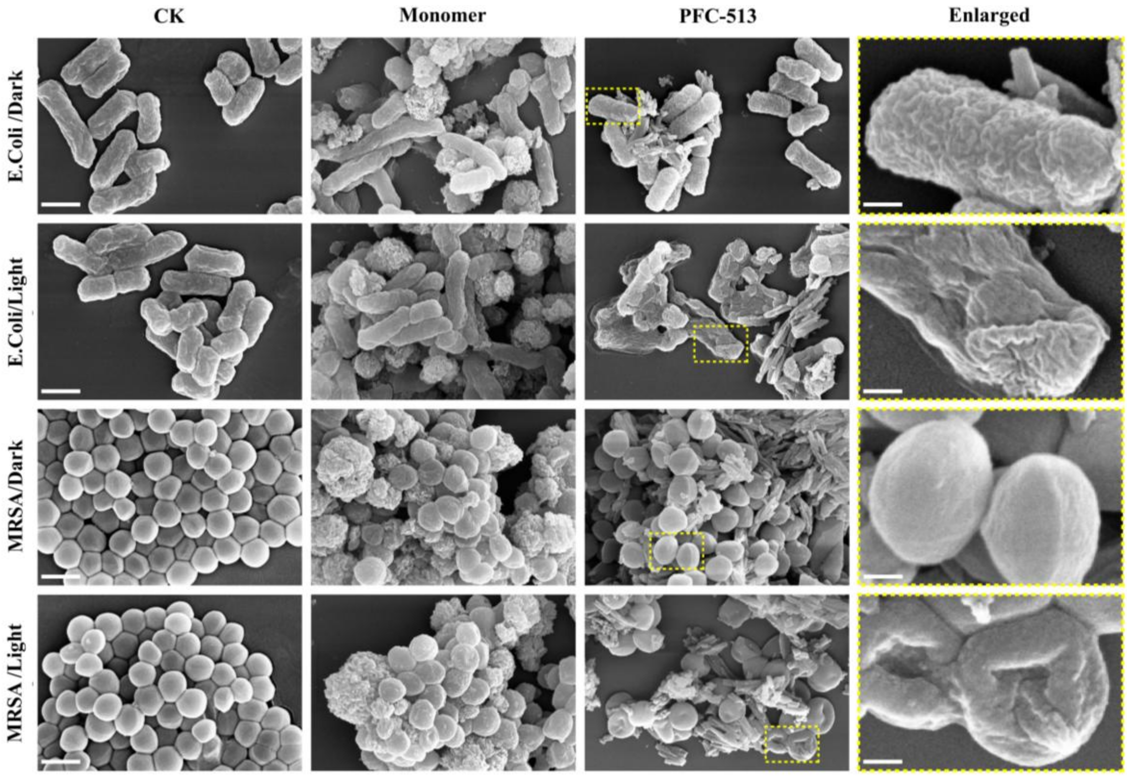

Figure S32. SEM image of bacteria after being treated in different condition (scale bar: 1 μm, 200 nm).

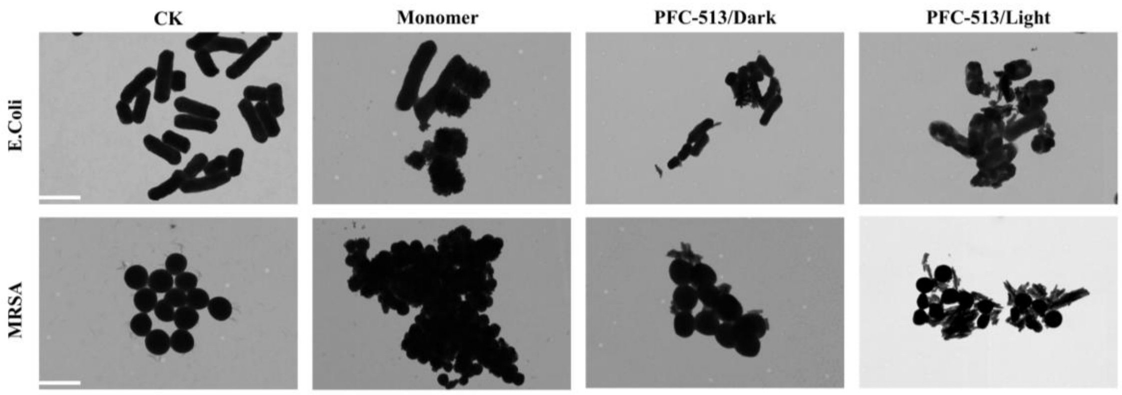

Figure S33. TEM image of bacteria after being treated in different condition. (scale bar: 1 μm).

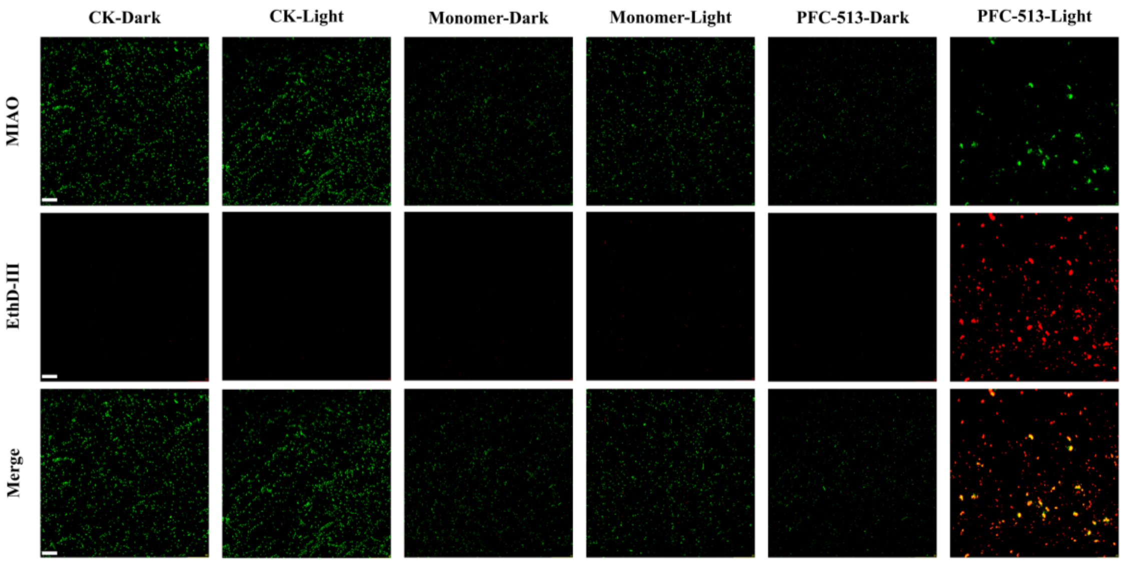

Figure S34. Live/Dead staining images of E. coli (scale bar: 40 μm).

2.20 Cell biocompatibility of PFC-513

HUVECs cells were seed in 96-well plates at a density of 5×10^3 cells per well and pre-cultured in an incubator for 24 h. PFC-513 at concentrations of 0, 10, 20, 30, 40 and 50 μg/mL were added, and cells were incubated for an additional 24 h. Afterwards, 10 μL of CCK-8 reagent (5 mg/mL) was added to each well, followed by a 30-minute incubation. The absorbance at 450 nm was measured using a microplate reader. Each concentration was tested in five parallel trials.

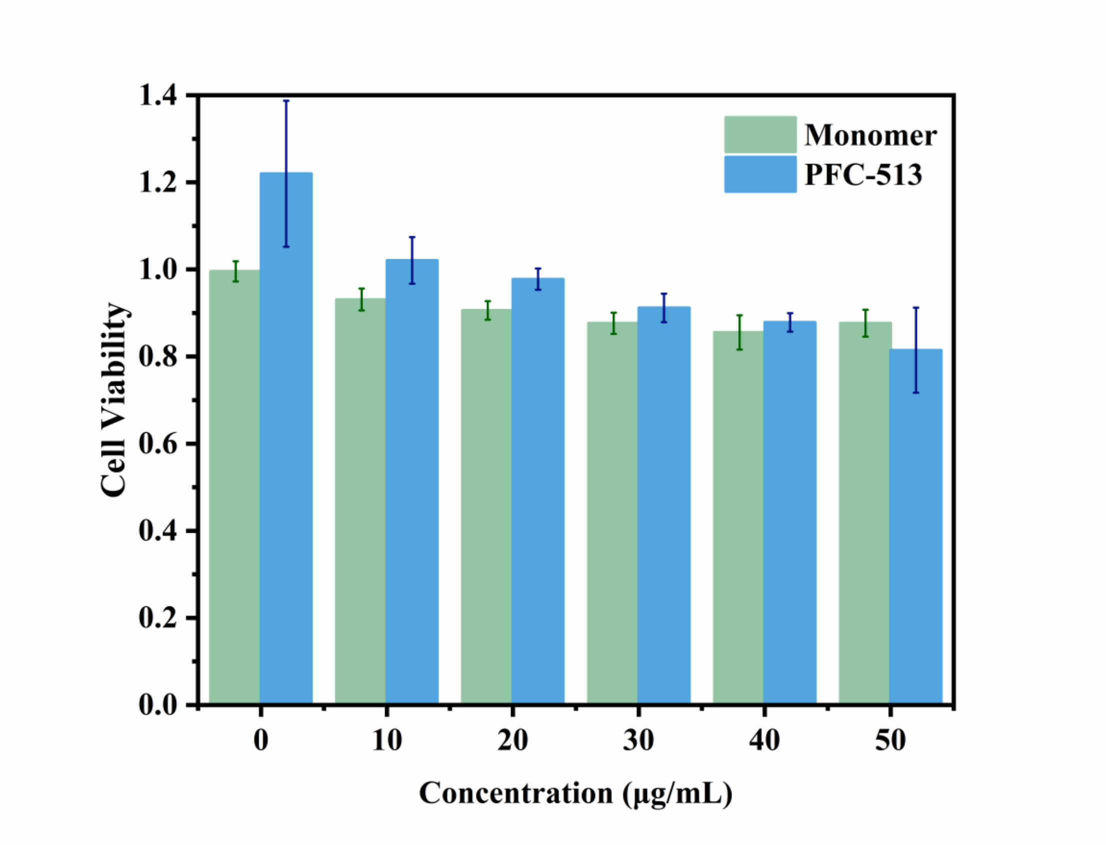

Figure S35. The CCK-8 assay of Monomer and PFC-513.

2.21 Biocompatibility of PFC-513[6]

All animal experiments were performed following the Guide and Care and Use of Laboratory Animals from the National Institutes of Health (NIH) and ARRIVE guideline, and approved by the Animal Care and Use Committees of Fujian Cancer Hospital, Fujian Medical University (NO. IACUC FJMU2025-Y-0767). The male mice were used in all the experiments. ICR mice were purchased from Shanghai Model Organisms Center. ICR mice (18-20 g) were administered with PFC-513 (10 mg/kg, saline) via tail vein injection and the control group was given an identical volume of saline. After administration for 24 hours, the mice were euthanized and the major organs, including the heart, liver, spleen, lungs, and kidneys, were isolated. These organs were then fixed, paraffin-embedded, cut into slices, and stained with hematoxylin and eosin (H&E).

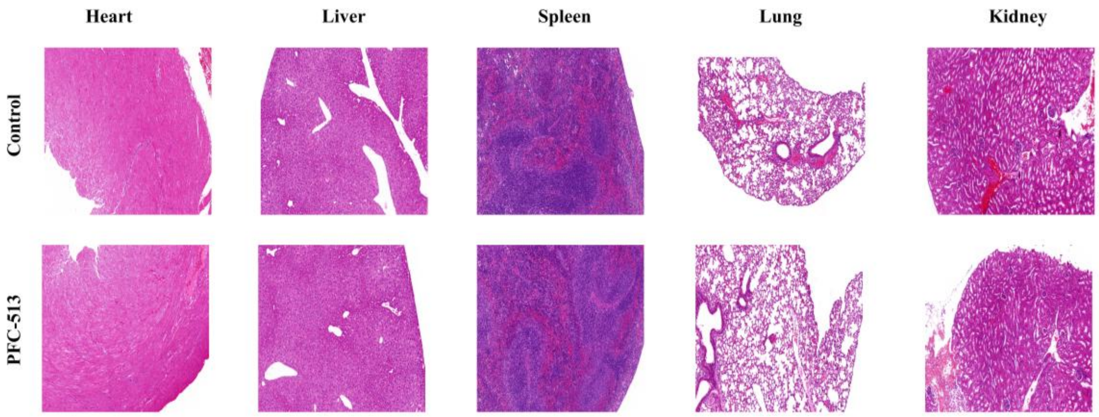

Figure S36. Histological sections of the heart, kidney, liver, lung, spleen tissues by H&E staining in each group after 24 hours of treatment.

2.22 Hemolysis test of PFC-513

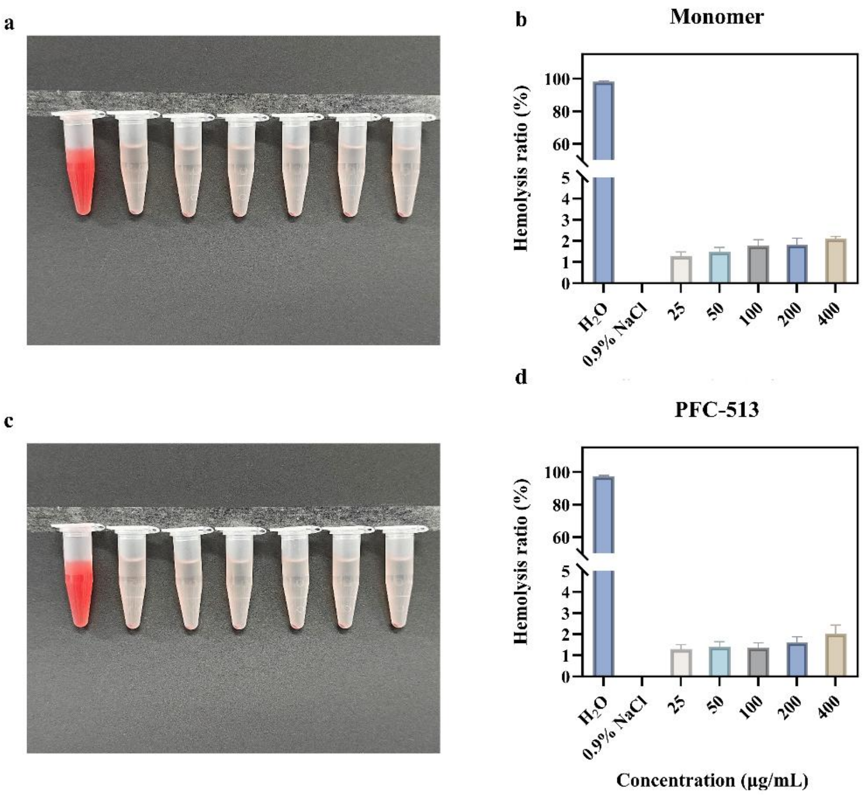

Figure S37. The hemolysis test and statistical analysis of a, b) Monomer and c, d) PFC-513.

2.23 Biostatistics of PFC-513

The diabatic rats bearing MRSA-infected skin wounds were randomly divided into four groups (n = 3 per group) and treated with Monomer and PFC-513. Wound debridement and dressing changes were performed at predetermined intervals, and the wound site was photographed and measured to calculate the wound healing rate. Hematoxylind and eosin (H&E) and Masson's trichrome staining were used to evaluate the healing process. Wound skin specimens were fixed with 10% formaldehyde, dehydrated with ethanol, and embedded in paraffin. Tissue sections (5 μm) were stained following standard protocols for H&E and Masson's trichrome staining. Briefly, the H&E staining protocol included sequential deparaffinization, an alcohol gradient pass, hematoxylin (0.5%) staining for 10 min, washing with 0.5% hydrochloric acid alcohol, and eosin (0.5%) staining for 10 min. The Masson's trichrome staining process included deparaffinization, an alcohol gradient pass, and sequential staining wit Masson blue, Reichun/magenta, and aniline blue. All stained sections were evaluated under an optical microscope.

For immunofluorescence staining of CD31, α-SMA, and COL1 at the wound sites, skin sections were first dewaxed and dehydrated. Antigen retrieval was processed under hydrothermal conditions, followed by blocking with a 3% H_2O_2/methanol solution. After blocking, 100 μL of goat serum was dropped to the section and incubated at RT for 20 min. Next, 100 μL of primary antibody (diluted 1:100, Abcam) was added dropwise and incubate at 37 °C for 3 h. The tissue section was then washed three times with PBS for 5 min each. The tissue was incubated with goat anti-rabbit IgG and goat anti-mouse IgG secondary antibodies corresponding to the primary antibodies. Cell nuclei were counterstained with DAPI, followed by additional PBS washes. Images were captured using a fluorescence microscope and quantitatively analyzed by ImageJ software.

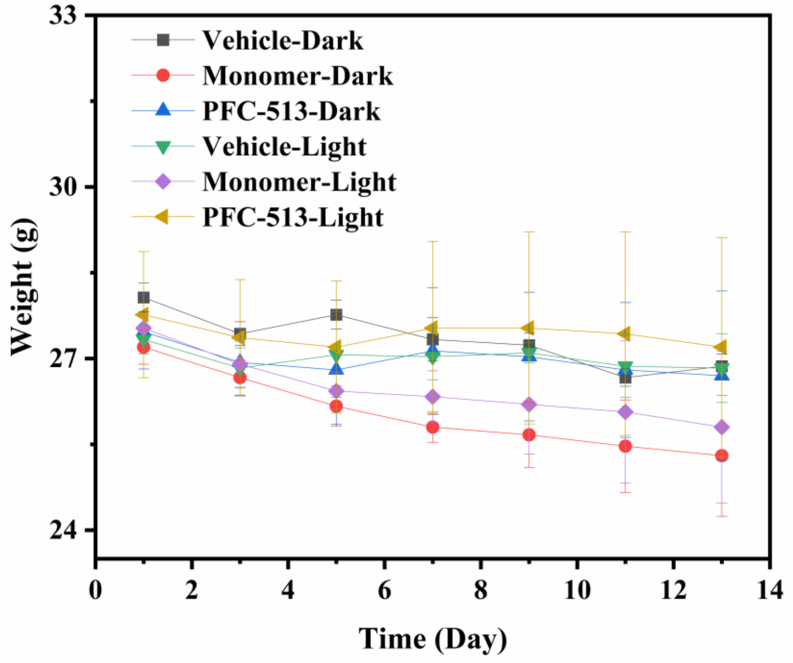

Figure S38. Mouse weight curves throughout the skin regeneration procedure, n=3.

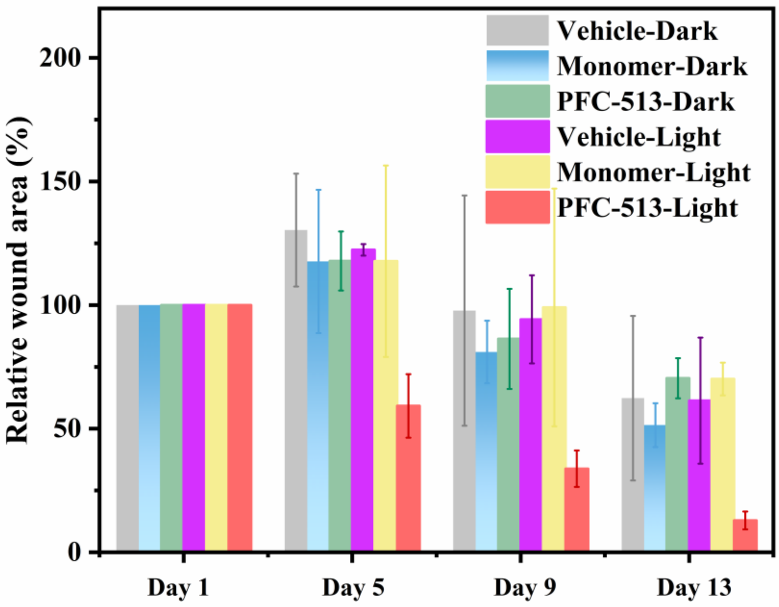

Figure S39. Wound healing time for each group, n=3.

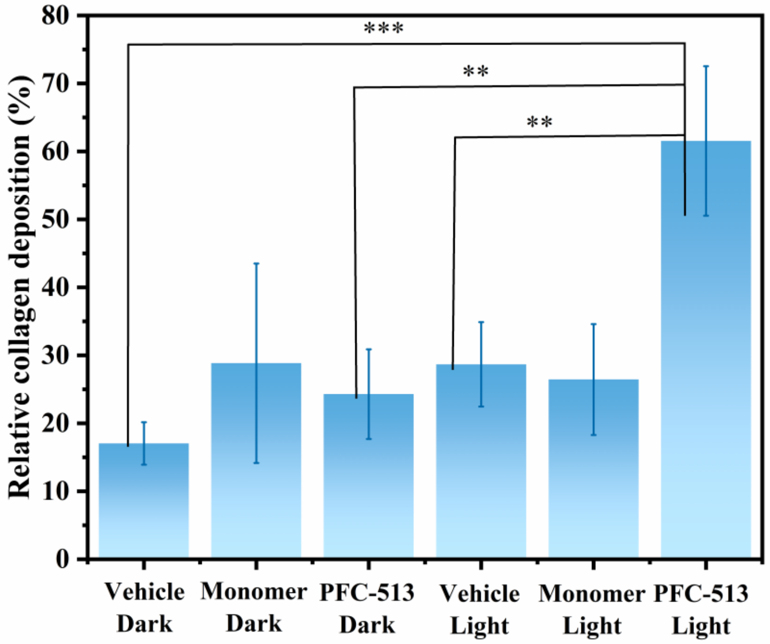

Figure S40. Quantitative analysis of the levels of collagen deposition from Masson images. The data were presented as the means ± SDs (n = 3) and were analyzed by one-way ANOVA with GraphPad Prism software. Statistical data are presented as mean ± SD (*$p < 0.05$, **$p < 0.01$, ***$p < 0.001$, ****$p < 0.0001$).

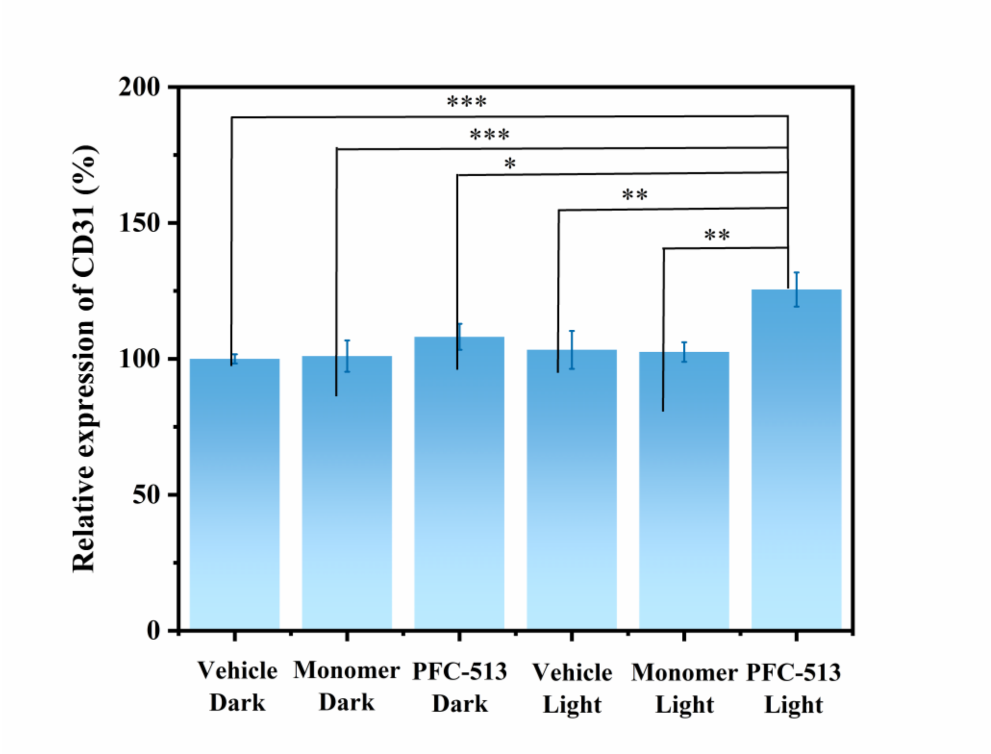

Figure S41. Quantitative analysis of the levels of CD31 from immunofluorescence images. The data were presented as the means ± SDs (n = 3) and were analyzed by one-way ANOVA with GraphPad Prism software. Statistical data are presented as mean ± SD (*$p < 0.05$, **$p < 0.01$, ***$p < 0.001$, ****$p < 0.0001$).

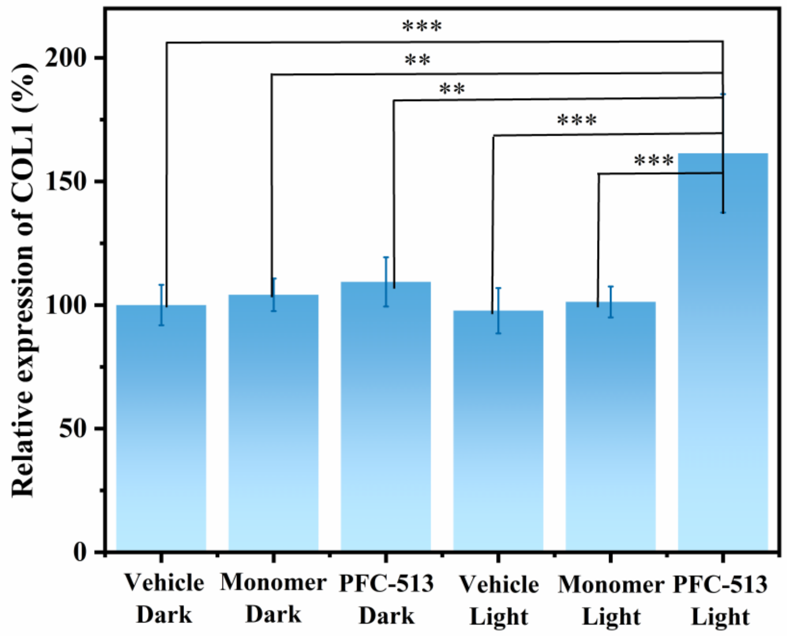

Figure S42. Quantitative analysis of the levels of COL1 from immunofluorescence images. The data were presented as the means ± SDs (n = 3) and were analyzed by one-way ANOVA with GraphPad Prism software. Statistical data are presented as mean ± SD (*$p < 0.05$, **$p < 0.01$, ***$p < 0.001$, ****$p < 0.0001$).

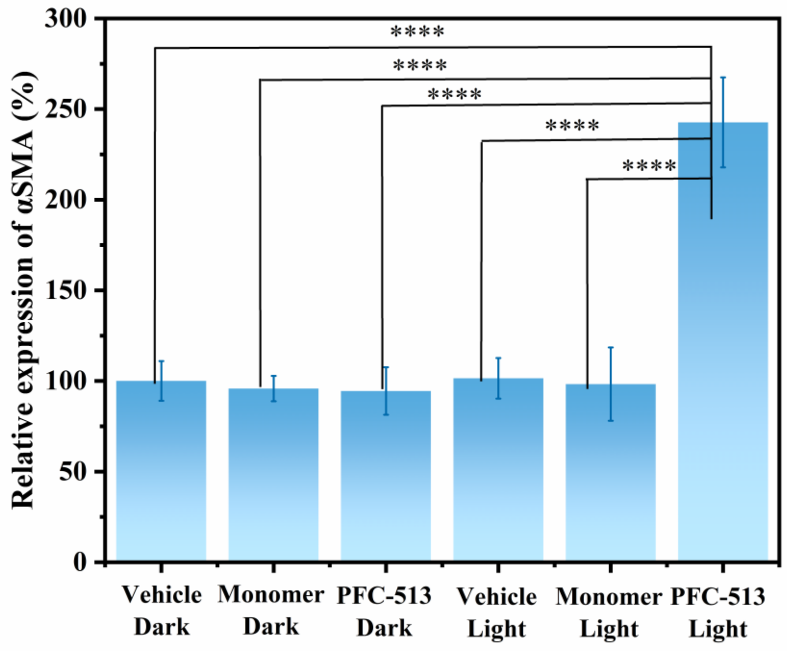

Figure S43. Quantitative analysis of the levels of αSMA from immunofluorescence images. The data were presented as the means ± SDs (n = 3) and were analyzed by one-way ANOVA with GraphPad Prism software. Statistical data are presented as mean ± SD (*$p < 0.05$, **$p < 0.01$, ***$p < 0.001$, ****$p < 0.0001$).

2.24 RNA sequencing (RNA-seq) analysis

Total RNA was extracted from the tissue using TRIzol® Reagent according to the manufacturer's instructions (Invitrogen) and genomic DNA was removed using DNase I (TaKara). Then RNA quality was determined by 2100 Bioanalyser (Agilent) and quantified using the ND-2000 (NanoDrop Technologies). RNA purification, reverse transcription, library construction and sequencing were performed at Shanghai Majorbio Bio-pharm Biotechnology Co., Ltd. (Shanghai, China) according to the manufacturer's instructions (Illumina, San Diego, CA). The mice skin RNA-seq transcriptome library was prepared following Illumina® Stranded mRNA Prep, Ligation from Illumina (San Diego, CA) using 1μg of total RNA. The raw paired-end reads were trimmed and underwent quality control by SeqPrep and Sickle using default parameters. The data were analyzed online (www.majorbio.com). All 6 files of transcriptome RNA-seq data (SRA) have been uploaded to NCBI and the BioProject ID is PRJNA1293773.

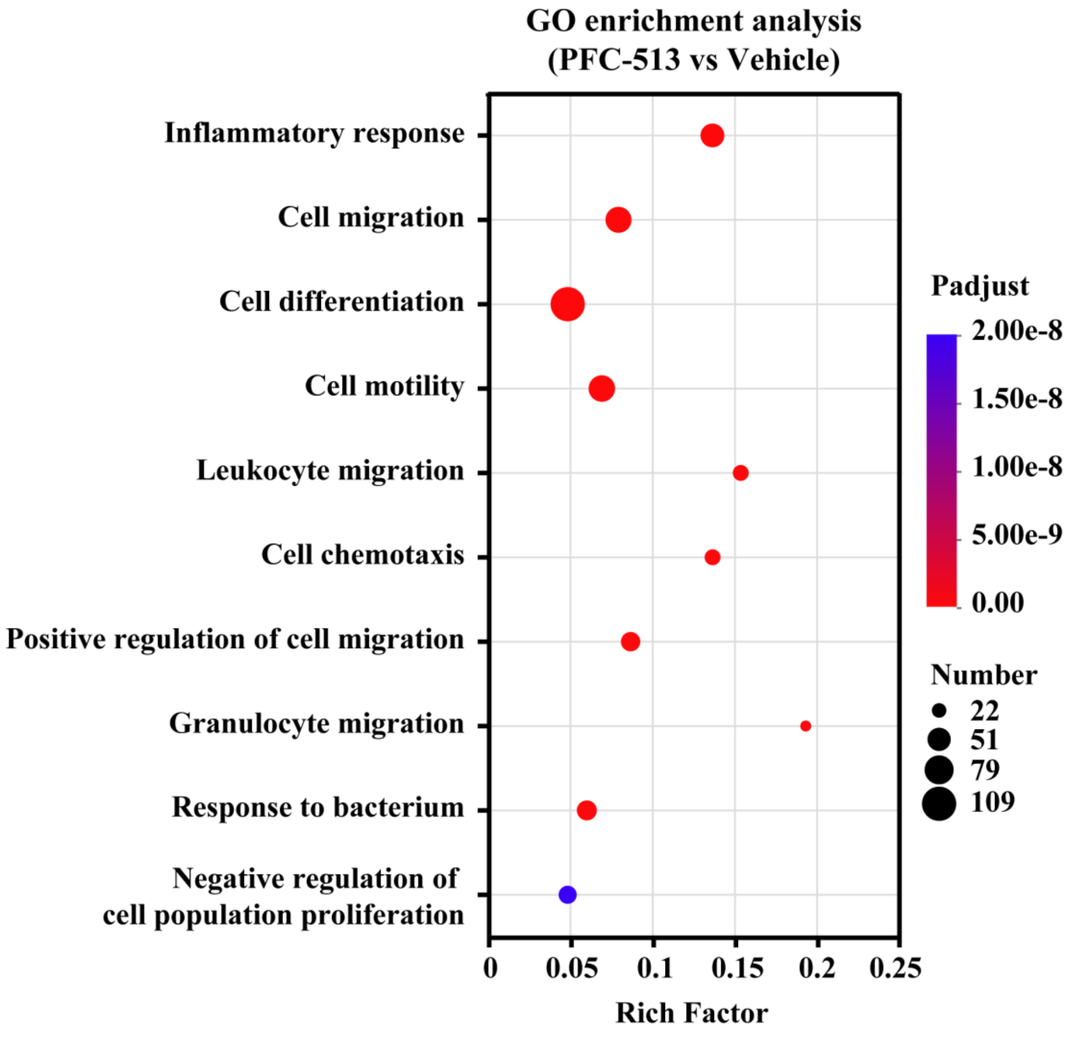

Figure S44. Genetic ontological (GO) analysis of differential genes in PFC-513 treatment groups.

Supplemental References

[1] A. Mallick, H. Liang, O. Shekhah, J. Jia, G. Mouchaham, A. Shkurenko, Y. Belmabkhout, H. N. Alshareef, M. Eddaoudi, *Chemical Communications* **2020**, *56*, 1883–1886.

[2] J. Q. Chen, K. Y. Zhang, X. D. Zhang, Z. Q. Huang, H. Deng, Y. Zhao, Z. Z. Shi, W. Y. Sun, *Chemistry – A European Journal* **2024**, *30*.

[3] H. X. Liu, D. H. Si, M. F. Smith, R. F. Li, X. Y. Li, L. Li, H. B. Huang, Z. B. Fang, H. C. Zhou, T. F. Liu, *Aggregate* **2023**, *4*.

[4] Y. Zou, H. X. Liu, L. Cai, Y. H. Li, J. S. Hu, C. Liu, T. F. Liu, *Advanced Materials* **2024**, *36*.

[5] B. T. Liu, X. H. Pan, D. Y. Zhang, R. Wang, J. Y. Chen, H. R. Fang, T. F. Liu, *Angew Chem Int Edit* **2021**, *60*, 25701–25707.

[6] Y. Li, Y. Li, S. Xu, Y. Chen, P. Zhou, T. Hu, H. Li, Y. Liu, Y. Xu, J. Ren, Y. Qiu, C. Lu, *Pharmacol. Res.* **2022**, *185*, 106491
